# Precursor Development and Aerosol‐Assisted Chemical Vapour Deposition for BiVO_4_ and W‐Doped BiVO_4_ Photoanodes: A Universal Ligand Approach

**DOI:** 10.1002/cssc.202401452

**Published:** 2024-11-08

**Authors:** Thom R. Harris‐Lee, Matthew K. Surman, Andrew J. Straiton, Frank Marken, Andrew L. Johnson

**Affiliations:** ^1^ Department of Chemistry University of Bath Claverton Down Bath BA2 7AY UK; ^2^ School of Chemistry Monash University Clayton, Vic 3800 Australia

**Keywords:** Photo-electrochemistry, Water splitting, BiVO_4_, Doping, AACVD

## Abstract

Green hydrogen production is a key area of importance for advancing into a completely sustainable world, not only for its use in industry and ammonia production, but also for its potential as a new fuel. One promising method for generating green hydrogen is light‐driven water splitting using photoelectrodes. Here, a bismuth vanadate (BiVO_4_) photoanode deposition process was developed using new, bespoke dual‐source precursors, tailored for use in aerosol‐assisted chemical vapour deposition (AACVD). The resulting thin films were highly nanostructured and consisted of phase‐pure monoclinic BiVO_4_. Pristine films under 1 sun solar irradiation yielded photocurrent densities of 1.23 mA cm^−2^ at 1.23 V vs RHE and a peak incident photon‐electron conversion efficiency (IPCE) of 82 % at 674 nm, the highest performance of any CVD‐grown BiVO_4_ film to date. A new, AACVD‐compatible WO_3_ precursor was subsequently designed and synthesised for the deposition of W‐doped BiVO_4_ within the same single deposition step.

## Graphical abstract

Here, a bismuth vanadate (BiVO_4_) photoanode deposition process has been developed using new, dual‐source precursors, tailored for use in aerosol‐assisted chemical vapour deposition (AACVD). The resulting thin films are highly nanostructured and consisted of phase pure monoclinic BiVO_4_. Pristine films under 1 sun solar irradiation yielded photocurrent densities of 1.23 mA cm^−2^ at 1.23 V vs RHE and a peak incident photon‐electron conversion efficiency (IPCE) of 82 % at 674 nm, the highest performance of any CVD‐grown BiVO_4_ film to date.

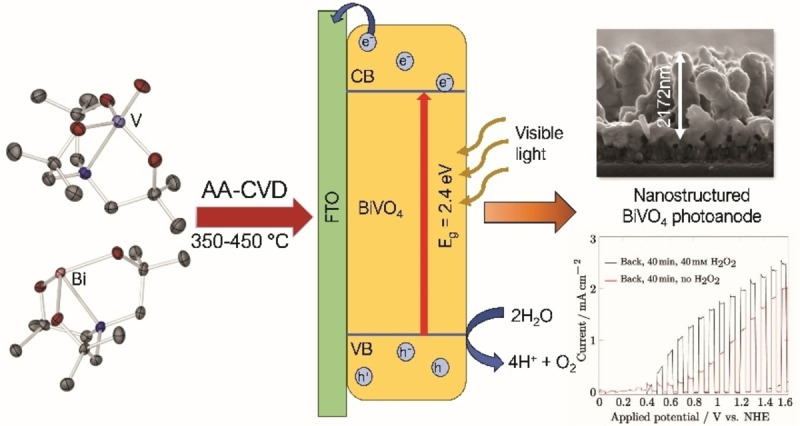

## Introduction

Hydrogen generation without the use of fossil fuels is a key area of importance for advancing into a completely sustainable world; not only is hydrogen essential for its use in industry and ammonia production, but also has great potential as a new fuel for long‐term energy storage.^[1]^ Photoelectrochemical (PEC) water splitting is an area of high interest as a production method for cheap, sustainable, and green hydrogen. The main limitation of the technique to date is the development of viable photoanode materials that meet the requirements not only for performance in hydrogen production, but also device scalability, stability, cost‐effectiveness, and earth‐abundant metal compositions.[^2^, ^3^, ^4^]

Aerosol‐assisted chemical vapour deposition (AACVD) is a subset of the chemical vapour deposition (CVD) technique, whereby precursors are dissolved into a solvent which is aerosolised and thermally decomposed over a heated substrate.^[5]^ AACVD is a highly scalable deposition method that has been applied to produce a wide range of thin film semiconductors and, significantly, can be easily expanded to the deposition of complex doped and/or mixed‐metal materials due to the lack of strict restrictions on precursor design and properties compared to vaporisation‐based CVD techniques.[^6^, ^7^, ^8^]

Alongside titanium dioxide (TiO_2_) and iron oxide (Fe_2_O_3_),[^9^, ^10^, ^11^] bismuth vanadate (BiVO_4_) is one of the standout candidates for use in photoanodes, owing to its low production cost, high stability, low toxicity, and narrow band gap (2.4–2.5 eV).^[12]^ Furthermore, the conduction and valence band edges are well placed relative to both hydrogen and oxygen evolution potentials.^[3]^ It is commonly fabricated through the simultaneous deposition of mixed dual‐source precursors, one bismuth (Bi) and one vanadium (V) based complex,[^6^, ^13^] since single‐source precursors are more restricted in design and often contain potential contaminants.^[14]^ It is not so common for these precursors to be novel, nor designed specifically for effective use with each other and for a specific deposition technique. Tungsten oxide (WO_3_) is commonly coupled with BiVO_4_ due to the WO_3_ conduction band minima sitting below that of BiVO_4_, facilitating electron injection into the WO_3_ and enhanced charge separation.[^15^, ^16^, ^17^]

The concept of a “universal” ligand system in CVD, which involves the design of a common framework capable of binding to metal ions, has the potential to serve as the basis of a universal precursor toolkit, providing molecular‐level control of precursors. Prime examples of this concept can be found across catalysis and CVD.[^8^, ^18^, ^19^, ^20^, ^21^] However, it should be noted that while a universal precursor may provide a common ligand set which allows for a shared structural form, and thus a common set of physical parameters such as solubility, thermal stability, and decomposition pathway, its use in itself does not affect quality of deposition in a CVD process. To design and develop just such a ‘universal’ ligand set which allows for the simple and efficient production of precursors with common attributes, we have developed amino tris‐alcohols as a tuneable pre‐ligand set.

**Figure 1:**
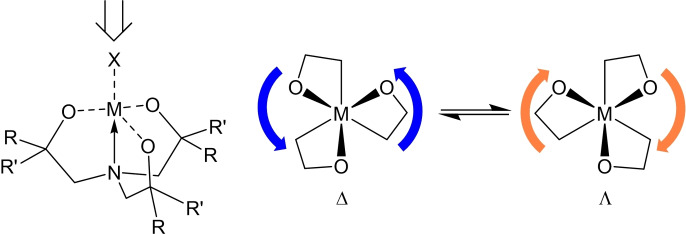
Δ and Λ stereochemistry for metallatranes viewing along the X−M−N axis (X is omitted for clarity).

Metal or metalloid‐derivatives of triethanolamines, known as metallatranes,[^22^, ^23^, ^24^, ^25^, ^26^] have been extensively studied and expanded across the periodic table, and are characterized by their cage‐like structure, comprising of an intramolecular transannular N→M interaction from a bridgehead N atom in the amine‐triethanolate ligand to a metal atom (M=metal). The N→M interaction results in a displacement or tilting in the same direction of the ligand framework, thereby making each of the chelating rings puckered and the resulting complex a three‐bladed propeller‐like stereo‐structure the chirality of which, is described in terms of right‐ or left‐handed (Δ or Λ) propeller geometry (Figure 1). and metallatranes.

While metallatranes have been reported for a number of metals,[^22^, ^23^, ^26^, ^27^] it is only recently that we have described the application of titanatrane complexes in the formation of TiO_2_ precursors.^[9]^ As complexes metallatranes (Figure 1), possess a tripodal, tetradentate‐(O_3_N) ligand framework serves to tailor the coordination environment of the metal atom, resulting in either monomeric or dimeric complexes in the solid state (where R and R’ ≠ H). As ligands, the judicious use of selected oxirane derivatives allows the steric and electronics of these ligand systems, to be tuned to some degree. As such a wide range of ligands and complexes can be formed.

In continuation of our studies on metallatranes as precursors for AACVD, we herein report the synthesis of a series of new, bespoke Bi and V complexes bearing amine tris‐alkoxide ligands. These metallatrane systems can be used as dual‐source precursors to grow phase‐pure, highly nanostructured, monoclinic BiVO_4_ by AACVD, followed by the inclusion of a complementary WO_3_ precursor to deposit W‐doped BiVO_4_ thin films. This is one of a small number of reported cases of BiVO_4_ grown by AACVD, and significantly shows a high photocurrent density of 1.23 mA cm^−2^ at 1.23 V vs RHE (V_RHE_).[^13^, ^28^] A complementary W‐based precursor has been used to dope AACVD‐grown BiVO_4_ for the first time, and significantly within the same single‐step deposition process as the undoped BiVO_4_ film.

## Results and Discussion

**Figure 2:**
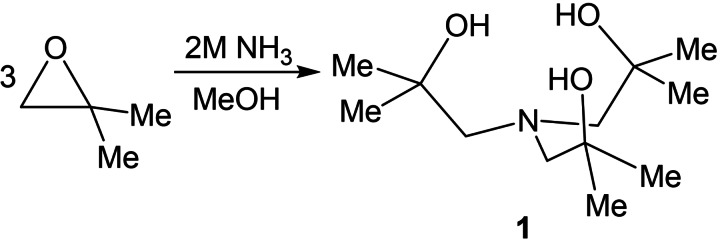
Synthetic procedure for tripodal amino alcohol ligands, procedure modified from work by Kim *et al*.^[30]^

Reaction of 1,2‐epoxy‐2‐methylpropane in a 3 : 1 reaction with ammonia in methanol results in the formation of the tris‐aminoalcohol, **1** (Figure 2). **1** was fully characterized by ^1^H and ^13^C^1^H NMR spectroscopy, displaying well‐defined resonances with expected integrations (see section 4.3).

**Figure 3:**
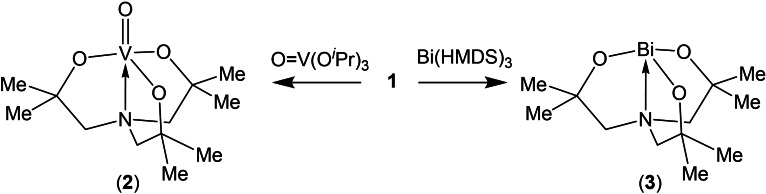
Synthetic procedure for the vanadyl and bismuth precursors **2** and **3**.

Reaction of the pro‐ligand **1**, with either V(O)(O^*i*^Pr)_3_ or Bi(HMDS)_3_ results in the formation of the vanadyl and bismuth precursors **2** and **3**, respectively, as shown in Figure 3. Both complexes characterised by ^1^H and ^13^C^1^H NMR spectroscopy, elemental analysis, and thermogravimetric analysis and are consistent with the presence of C_3_ symmetric complexes in the solution state.

**Figure 4:**
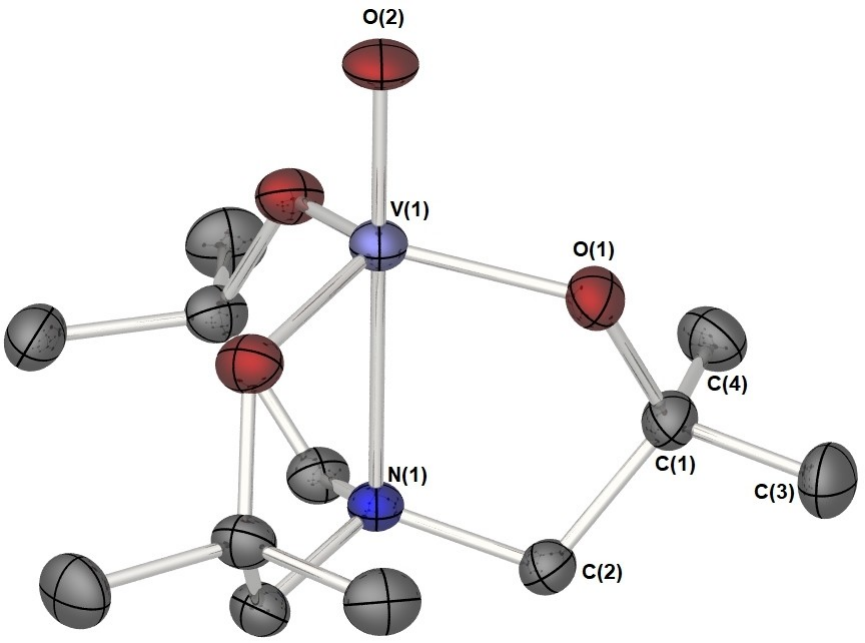
Solid state molecular structure of the vanadium oxo system **2**. Thermal ellipsoids are shown and 50 %, hydrogen atoms have been omitted for clarity. Selected bond lengths (Å) and angles (°): V(1)‐O(1) 1.7995(18), V(1)‐O(2) 1.627(5), V(1)‐N(1) 2.356(5): O(1)‐V(1)‐O(2) 101.88(6), N(1)‐V(1)‐O(2) 180.0, O(1)‐V(1)‐O(1 A) 115.88(4), O(1)‐V(1)‐N(1) 78.12(6). Symmetry transformations used to generate equivalent atoms: #A ‐y+1,x‐y,z and #B ‐x+y+1,–x+1,z.

**Figure 5:**
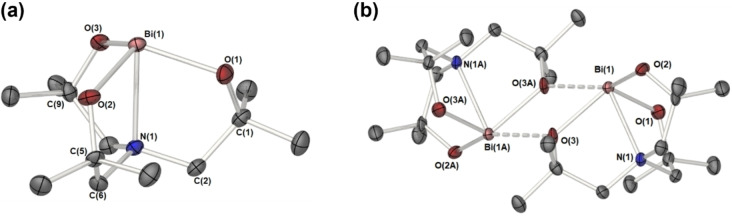
Solid state molecular structure of the bismuth monomer system **3** (A) and the dimeric unit (B). Thermal ellipsoids are shown and 50 %, hydrogen atoms have been omitted for clarity. Selected bond lengths (Å) and angles (°): Bi(1)‐O(1) 2.117(3), Bi(1)‐O(2), 2.161(3) Bi(1)‐O(3) 2.174(3), Bi(1)‐N(1) 2.524(4), Bi(1)‐O(3 A) 2.776(3); O(1)‐Bi(1)‐N(1) 73.29(11), O(2)‐Bi(1)‐N(1) 72.28(11), O(3)‐Bi(1)‐N(1) 71.85(10), O(1)‐Bi(1)‐O(2), 108.31(11), O(1)‐Bi(1)‐O(3) 110.85(11), O(2)‐Bi(1)‐O(3) 114.76(11), O(3)‐Bi(1)‐O(3 A) 46.64(10) Bi(1)‐O(3)‐Bi(1 A) 114.85(11). Symmetry transformations used to generate equivalent atoms: #A −1‐x, 1‐y,‐z.

The C_3_ symmetry of complexes **2** and **3** is preserved in the solid state: Complexes **2** and **3** each adopt a monomeric structure in the solid state, mainly due to the steric protection by the methyl groups of the atrane framework. Complex **2** crystallizes in the chiral hexagonal space group P6_3,_ with the central {O−V‐N} atoms of **2** lying on a crystallographic threefold axis such that symmetry operators generate a C_3_ symmetric molecule. Similarly, molecules of **3** possess approximate C_3_ symmetry, with the complex crystalising in the achiral monoclinic space group P2_1_/n with one complete molecule in the asymmetric unit cell. The solid‐state structures of compounds **2** and **3** are shown in Figure 4 and Figure 5, respectively.

The coordination environment about the vanadium centre in **2** is a distorted trigonal bipyramid with the N(1) and O(2) atoms occupying the axial positions and the three oxygen atoms of the tris‐aminoalkoxide ligand, O(1)–O(1 A)‐O(1B), in equatorial positions. The geometrical geometry index or structural parameter^[29]^ (*τ*=1.06) falls close to that of an ideal trigonal bipyramidal system, with the vanadium atom only slightly displaced from the plane *E*(O_equatorial_) (~0.37 Å) defined by the equatorial oxygen atoms in direction to the exocyclic ligand. Equatorial V−O bonds (1.627(5) Å), axial N→V distance (2.356(5) Å) and axial V=O (1.799(2) Å) distance are not unsurprisingly comparable to those in related systems.

To the best of our knowledge, complex **3** is the first example of a bismuth amine tris‐alkoxide complex, however, related bismuth nitrilotriacetate complexes have been reported.[^30^, ^31^] At first glance the bismuth atom appears to be four co‐ordinate with a stereochemically active lone pair, and the ligand adopting a tripodal, tetradentate, *C*_3_‐symmetric (non‐crystallographic), propeller‐like arrangement around the metal centre. The Bi atom itself lies ~1.20 Å above the plane of the three O atoms, resulting in a pseudo‐trigonal‐bipyramidal coordination geometry and a long (2.524(4) Å) N→Bi interaction. The Bi−O bond distances (average, 2.151 Å) are longer than those found in the simple homoleptic bismuth alkoxide [Bi(O^t^Bu)_3_] (average, 2.059 Å).^[32]^ A close examination of the data reveals intermolecular contacts between Bi(1) and O(3) atoms of a neighbouring complex of 2.776(3) Å, which while short compared to the sum of the van der Waals radii=4.04 Å (Bi; 2.54 Å, O: 1.50 Å), results in a symmetric dimer with a central {Bi_2_O_2_} core consisting of one long and one short Bi‐(μ‐0) bond, as noted for the other compounds. The asymmetric core leads to a Bi⋅⋅⋅Bi bond distance of 4.184 Å for **3**. The formation of the dimer increases the co‐ordination number of bismuth to five‐coordinate.

Tungsten is generally regarded as one of the best doping materials for BiVO_4_, enhancing any number of a wide range of PEC‐impacting material properties,^[33]^ hence an analogous tungsten oxide (WO_3_) precursor was developed and used to deposit W‐doped BiVO_4_ to improve overall device performance. A tungsten precursor was therefore synthesised using the same tris‐aminoalcohol proligand, **1**. It was hoped that by keeping the ligand system of the precursors consistent there would be some coherence between the deposition temperatures of the new tungsten precursor and the BiVO_4_ precursors **2** and **3** respectively, making it easy to integrate the tungsten dopant into the BiVO4 deposition process described in the previous section.

**Figure 6:**
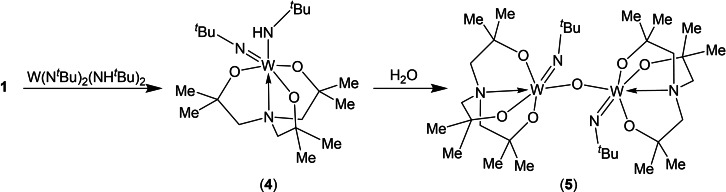
Synthetic procedure for the W precursor **5**.

To that end, the tris‐aminoalcohol, **1**, was reacted (1 : 1) with *bis*(*tert*‐butylimido)*bis*(*tert*‐butylamido)tungsten in THF. While multinuclear NMR data was consistent with the formation of complex **4**, as indicated by the presence of two different {^*t*^Bu} groups, two {CMe_2_} units and two broad resonances corresponding {CH_2_} groups. Attempts to isolate the complex pure was unsuccessful and successive attempts resulted in either formation of a pale yellow semi‐solid which contained a small number of crystals of the partial hydrolysis product, **5** (yield <5 %). Reaction of **4** with 1 equivalent of water in THF proceeds as shown in Figure 6, yielding **5** in good yields (>80 %) as a white crystalline solid, the ^1^H NMR spectrum of which is not dissimilar to that of **4**, the most significant change being the appearance of only one {^*t*^Bu} group. As with **4** the ^1^H NMR resonances of **5**, indicate a breaking of the C_3_ molecular symmetry observed in **2** and **3**. Attempt to react **4** with two equivalents of H_2_O, failed to yield higher oxo‐bridged species with **5** being isolated.

**Figure 7:**
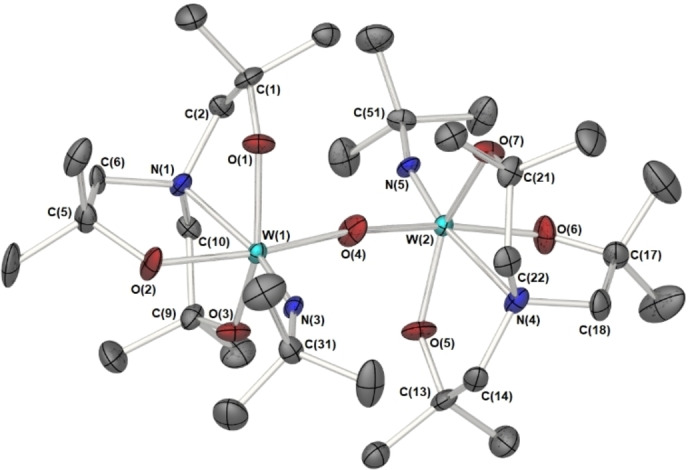
Solid state molecular structure of the tungsten oxo system 5. Thermal ellipsoids are shown and 50 %, hydrogen atoms and two molecules of toluene of crystallisation have been omitted for clarity. Selected bond lengths (Å) and angles (°): W(1)‐O(1) 1.968(4), W(1)‐O(2) 1.969(3), W(1)‐O(3) 1.931(4), W(1)‐N(1) 2.300(4), W(1)‐N(3) 1.753(3), W(1)‐O(4) 1.918(3) W(2)‐O(4) W(2)‐O(5) 1.942(4), W(2)‐O(6) 1.970(3), W(2)‐O(7) 1.952(4), W(2)‐N(4) 2.306(4), W(2)‐N(5) 1.752(3), 1.924(3): W(1)‐O(4)‐W(2) 169.63(19), N(1)‐W(1)‐N(3) 165.59(14), O(4)‐W(1)‐O(2) 170.53(12), O(3)‐W(1)‐O(1) 152.34(13), O(4)‐W(2)‐O(6) 169.17(14), O(5)‐W(2)‐O(7) 151.94(14), N(5)‐W(2)‐N(4) 166.65(14).

Complex **5** adopts a bi‐metallic structure in the solid state, centred about a {W−O‐W} core approaching linearity (169.63(19)°) as shown in Figure 7. Each tungsten atom adopts a distorted 6‐coordinate octahedral geometry. The molecular structure consists of two tungsten atoms bridged by a single oxo ligand, with further coordination about each metal by one {^*t*^BuN} and a chelating tris‐aminoalkoxide ligand (Figure 5). The bridge head‐nitrogen atom of the amine tris alkoxide ligand are found to lie trans to the imido {^*t*^Bu} group function where long N→W bond distances of 2.300(4) Å and 2.306(4) Å, for W(1)‐N(1) and W(2)‐ N(4) respectively, are observed and are considerably longer than the W=N imido bond distances of 1.753(3) Å and 1.752(3) Å, for W(1)‐N(3) W(2)–N(5) respectively. The W−O distance (average, 1.951 Å) are typical of W−O bond distances in a variety of tungsten alkoxide species.

Complete crystal data and structure refinement for **2**, **3**, and **5** is provided in section S4 (ESI). Thermal characterisation of **2**, **3**, and **5** has been undergone by thermal gravimetric analysis to reveal suitability for AACVD and compatibility for multi‐source depositions. The full results and discussion can be found in section S1 (ESI).

### BiVO_4_ Thin Films

The Bi and V precursors were used in a cold‐wall AACVD rig to deposit BiVO_4_ thin films. CVD of BiVO_4_ is commonly known to result in Bi‐rich impurity phases, partially because of poor V integration into the growing film.^[34]^ A common way to address this issue is to bias the process in favour of V. Deposition studies of compounds **2** and **3** therefore began with an investigation into the purity of BiVO_4_ that could be grown at 400 °C with precursor solutions containing different ratios of Bi and V precursors.

**Figure 8:**
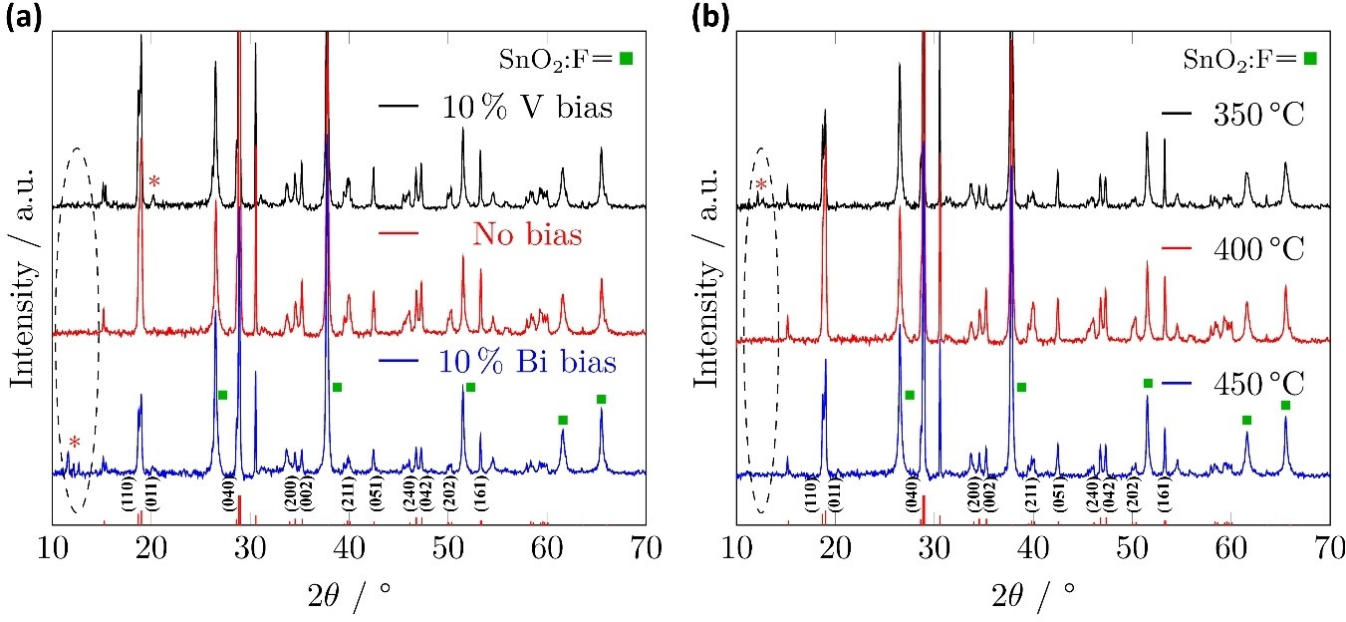
XRD patterns of BiVO_4_ films deposited for 30 min by aerosol‐assisted CVD of compounds **2** and **3** (a) at 400 °C using ratios of V to Bi precursors in a range of 1:0.8–0.8:, Black: V : Bi=1:0.8, red V : Bi=1 : 1, blue: V : Bi=0.8 : 1; (b) (V : Bi=1 : 1) at varying temperatures, black: 350 °C; red: 400 °C; blue: 450 °C. The area of interest for bismuth oxo‐cluster contamination has been highlighted with a black, dashed ellipse. A red asterix has been used to highlight impurity peaks. A standard distribution of major peak locations for m‐s BiVO_4_ have been marked with red lines directly along the bottom of the x‐axis.^[39]^

The diffraction peaks of BiVO_4_ at 2θ=15.1°(110), 19.0°(011), 30.5°(040), 34.5°(200), 35.8° (002), 39.8°(211), 42.5°(051), 47.3°(042), 50.3°(202), and 53.2°(161) can correspond to planes of monoclinic scheelite type BiVO_4_(JCPDS No.14‐0688).^[35]^ All three X‐ray diffraction (XRD) spectra obtained from BiVO_4_ films grown with V and Bi precursor ratios ranging from 1:0.8–0.8 : 1 (Figure 8a) are indicative of bulk monoclinic BiVO_4_, confirmed by the presence of a peak about 15° which is exclusive to the monoclinic phase.^[36]^ There was no indication of Bi‐rich impurities in the materials deposited from solutions with a V‐biased and non‐biased precursor solutions. The XRD pattern for the material produced from a 10 % V‐biased precursor solution, contains a peak about 20° (marked by a red asterisk), likely the {001} facet of V_2_O_5_,^[37]^ hence the excess V precursor was contributing to growth of V_2_O_5_. This, and the lack of impurity peaks in the non‐biased sample, is clear evidence that the integration of V into the BiVO_4_ was not hindered. As expected, by biasing the precursor solution in favour of Bi, Bi‐rich impurity peaks about 12° (appeared in the XRD pattern (marked by a red asterisk).^[38]^ These peaks were not present in non‐biased solutions, therefore a 1 : 1 ratio of Bi and V precursors was found to be the optimum solution composition to grow phase pure BiVO_4_ by AACVD.

To optimise the deposition temperature of compounds **2** and **3** for BiVO_4_ growth, precursor solutions with a 1 : 1 ratio of Bi to V precursor were deposited at temperatures ranging from 350–450 °C for 30 min, and subsequently analysed by X‐ray diffraction (XRD) (Figure 8b) to assess the crystallinity and purity of deposition. Notably, by reducin impedance spectroscopy active g the temperature to 350 °C, the XRD pattern evidenced Bi‐rich impurities, hence V incorporation into the growing film must be hindered at lower temperatures. This observation could in part be explained by the thermogravimetric analysis (TGA) data (Figure S2) which showed that the V precursor underwent a two‐step decomposition process, while the Bi precursor was volatile. The reduced temperature of 350 °C could both reduce V integration into the film through slower decomposition, whilst also increasing Bi incorporation by reducing repulsion of the volatile precursor from the substrate.

**Figure 9:**
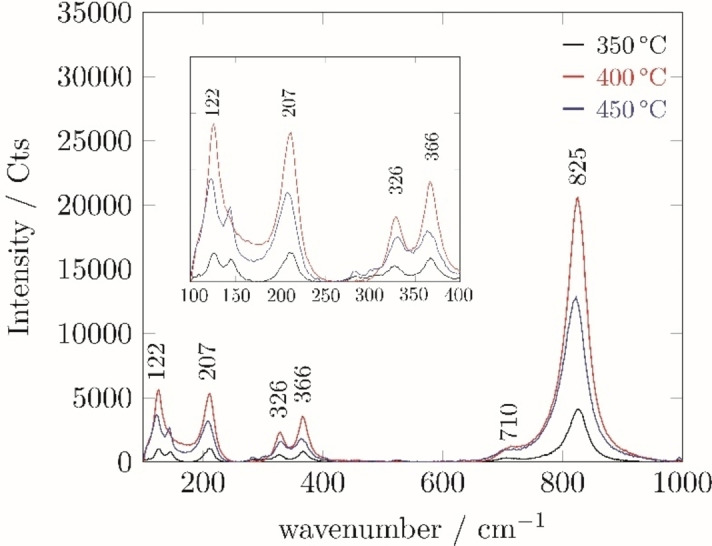
Raman spectra of BiVO_4_ films deposited by AACVD of compounds **2** and **3** at different temperatures for 30 min. Black: 350 °C; red: 400 °C; blue: 450 °C.

Notable differences in the XRD patterns at 2θ of 35, 40, 46, 50 and 60 can be seen in Figure 8b. The peaks for the 400 °C sample appeared to more accurately represent the expected XRD pattern, compared to those at 450 °C. The Raman spectra of the BiVO_4_ films deposited between 350–450 °C (Figure 9) were also all indicative of the monoclinic polymorph of BiVO_4_.^[40]^ As a result of all discussed analysis, optimised depositions were determined to be at 400 °C using a 1 : 1 molar ratio of Bi and V precursors.

The XRD patterns corresponding to the films deposited at both 400 and 450 °C did not contain Bi‐rich impurity peaks, therefore pure BiVO_4_ could be deposited within this temperature range. The identity of the thin films as BiVO_4_ was further validated by EDX of an as deposited thin film (400 °C using a 1 : 1 ratio of Bi and V precursors), with an At% averaged over five positions to be 0.7:0.6 : 1, indicative of an equal amount of bismuth and vanadium in the film. However, this also suggestive of a thin film which is oxygen‐deficient. It should also be noted that many EDX spectra reported for BiVO_4_ in the literature have also reported an oxygen deficiency.[^41^, ^42^, ^43^]

**Figure 10:**
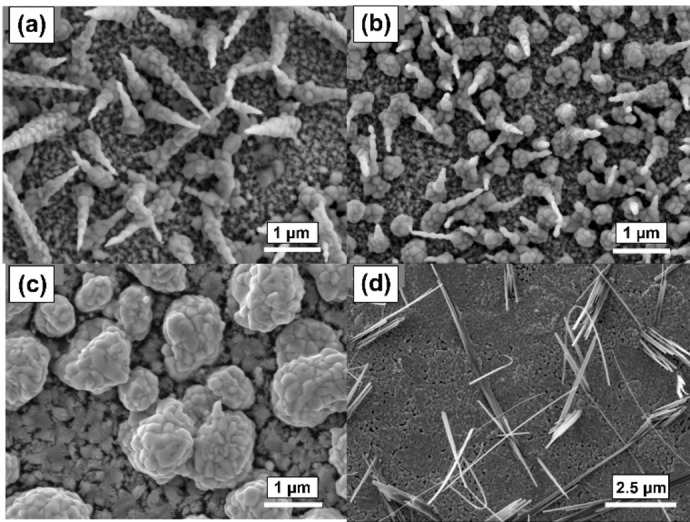
Top‐down electron micrographs of BiVO_4_ films deposited using compounds **2** and **3** with deposition process times of (a) 5 min, (b) 10 min, (c) 20 min, (d) 40 min.

**Figure 11:**
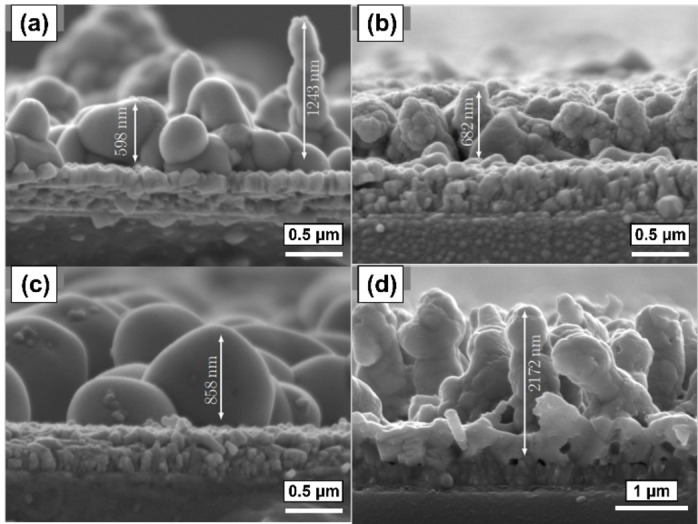
Cross sectional electron micrographs of BiVO_4_ films deposited using compounds **2** and **3** using deposition process times of (a) 5 min, (b) 10 min, (c) 20 min, (d) 40 min.

Using this optimised process, monoclinic BiVO_4_ films were grown on FTO substrates by AACVD in THF at 400 °C with deposition periods of 5, 10, 20 and 40 min to investigate the material growth. Top‐down and cross‐sectional scanning electron microscopy (SEM) images (Figures 10 and 11, respectively) were acquired on annealed films (540 °C, 2 hours, ambient air). The initial stages of growth of the monoclinic BiVO_4_ exhibited a cone‐like nanostructure, however at a deposition time of 20 min, the density of the cones had increased such that larger aggregates were formed during the annealing process; and by 40 min a clear, dense base layer had been formed, from which nanorod clusters were seen to extend. The 40 min top‐down image (Figure 10d) showed porosity resulting from the cone‐like nanostructure had been maintained, confirmed by cross‐sectional images which also more clearly showed the formation of 2 μm long BiVO_4_ pillars connected to the approximately 100 nm denser layer beneath. The resulting high surface area nanostructure is promising for a high performing photoanode.

Strangely, the cross‐sectional micrographs showed a relatively constant overall film thickness for the first 20 min of deposition, on average measuring from 598–858 nm. The thickness of the material then increased more rapidly from 858 to 2172 nm between 20 and 40 min, hence nucleation across the substrate surface must be slow, but with a significantly greater growth rate once full FTO surface coverage is achieved.

**Figure 12:**
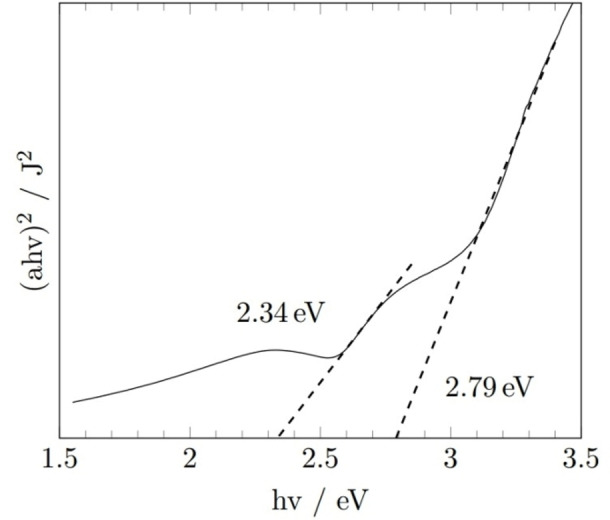
Tauc plot of the BiVO_4_ films deposited using compounds **2** and **3** at 400 °C. Photocurrent density was measured over a range of voltages during exposure to simulated solar light (AM 1.5 G, 100 mW cm^−2^) to evaluate the thin film performance for light‐driven water splitting. BiVO_4_ commonly performs better under rear‐side illumination than front‐side due to poor electron mobility and surface catalysis. As expected, the BiVO_4_ photoanodes here also produced significantly higher photocurrents under rear‐side illumination.

The bandgap of the BiVO_4_ film was obtained using UV/Vis spectroscopy absorbance measurements to produce a Tauc plot (Figure 12).^[44]^ Bandgap approximations of 2.34 eV and 2.79 eV were ascribed to the indirect and direct bandgaps energies of monoclinic BiVO_4_, with both values in agreement with previous reports of experimentally measured values.[^14^, ^45^, ^46^] It should be noted that the reported bandgap of Bi_2_O_3_ is within the range of 2.59–3.09 eV and 2.47–3.4 eV for the alpha and beta phases respectively,^[47]^ hence the 2.79 eV bandgap could be associated with some Bi_2_O_3_ impurity phases located within the film, however earlier XRD and Raman data confirmed pure BiVO_4_.

The largest photocurrent density for front‐side illumination was seen in the 5 min sample, with a subsequent trend of decreasing performance with increased deposition time. Front illumination generates a greater number of electron‐hole pairs closer to the electrode‐electrolyte interface, so on average electrons must migrate further to the back contact, hence increasing charge recombination. When carrier transport through the material is a limiting factor, a common issue of BiVO_4_, a thicker film increases this source of charge recombination, resulting in lower measured photocurrent. When illuminated from the rear, the samples deposited for 5, 10 and 20 min all produced similar photocurrent densities of around 0.10 mA cm^−2^ at 0.8 V_RHE_ (highest voltage under which BiVO_4_ is theoretically stable) and 0.44 mA cm^−2^ at 1.23 V_RHE_, as expected given the similar thicknesses between films. The 40 min sample, however, produced 0.39 and 1.23 mA cm^−2^ at 0.8 and 1.23 V_RHE_ respectively, this significant increase likely due to the substantially increased sample thickness from 20 to 40 min depositions, as well as the increased surface area for enhanced light absorption and surface charge transfer.

**Figure 13:**
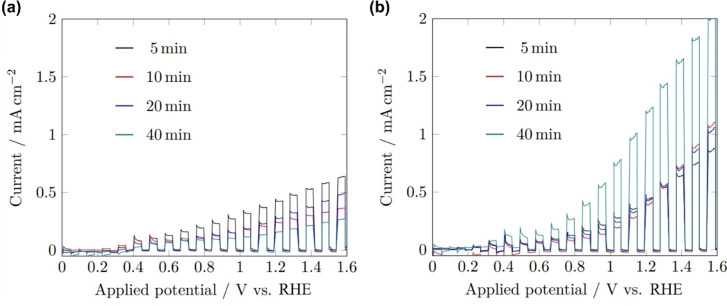
Linear sweep voltammograms of BiVO_4_ films deposited for 5 (black), 10 (red), 20 (blue) and 40 min (teal) under 1 sun chopped (5 s on/off) (a) front‐side, (b) rear‐side illumination (AM 1.5 G, 100 mW cm^−2^). All measurements performed in 1 M buffered potassium phosphate electrolyte (pH 6.6) with a 10 mV s^−1^ scan rate.

Surprisingly, the films reported in these works were seen to produce a photocurrent across the range 0–1.6 V_RHE_, in comparison to the onset potentials of AACVD‐grown BiVO_4_ reported previously of 0.6 V_RHE_ and 0.33 V_RHE_.[^13^, ^28^] The onset potential of a photoelectrode reflects the hole transfer kinetics between the electrode and the electrolyte, indicating that the surface kinetics of the BiVO_4_ grown here were superior, likely as a result of the increased relative detection of {010} facets seen in the XRD measurements, facets known to improve water‐splitting performance (Figure 13).^[48]^

**Figure 14:**
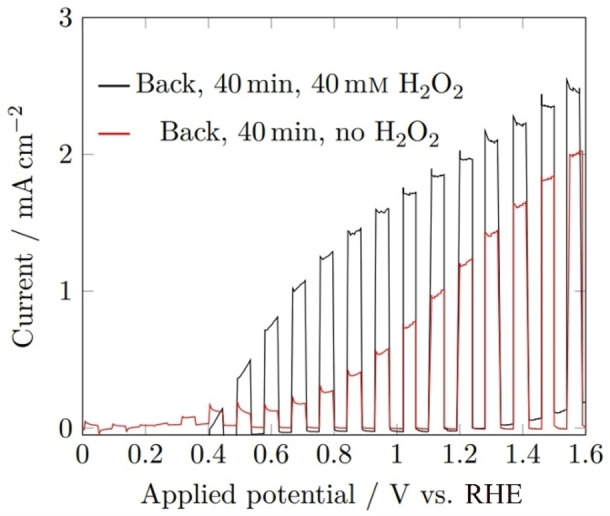
Linear sweep voltammograms of BiVO_4_ films deposited for 40 min under 1 sun chopped (5 s on/off) rear illumination (AM 1.5 G, 100 mWcm^−2^), performed in 1 M buffered potassium phosphate electrolyte (pH 6.6) with 40 mM H_2_O_2_ (black), no H_2_O_2_ (red); using a scan rate of 10 mV s^−1^.

The photocurrent density was significantly larger in the presence of a hole scavenger, H_2_O_2_, (Figure 14) increasing from 1.23 to 1.97 mA cm^−2^ at 1.23 V_RHE_, and from 0.39 to 1.29 mA cm^−2^ at 0.8 V_RHE_, hence the overall performance was still significantly hindered by the charge transfer kinetics at the electrode‐electrolyte interface. It is therefore necessary to deposit a co‐catalyst onto the BiVO_4_ surface for future studies to overcome the limited surface kinetics, particularly at lower voltages.

**Figure 15:**
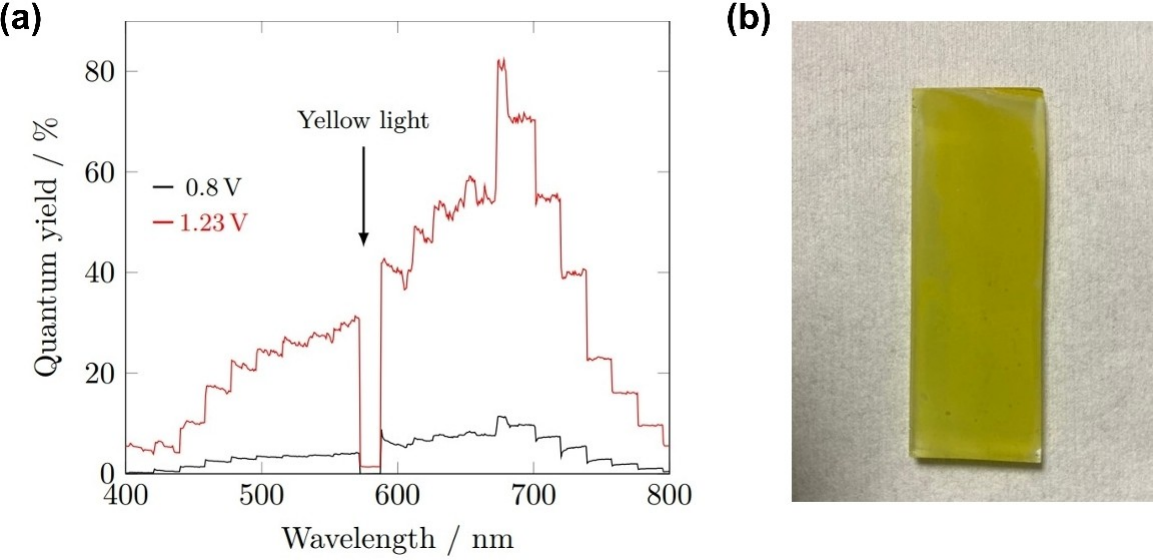
(a) IPCE spectra for undoped BiVO_4_ films at 0.8 V_RHE_ (black) and 1.23 V_RHE_ (red), measurements performed in 1 M buffered potassium phosphate (pH 6.6); (b) Photograph of the as‐deposited BiVO_4_ films, showing the yellow colouration.

The incident photon‐electron conversion efficiency (IPCE) of the 40 min BiVO_4_ sample was calculated at both 0.8 and 1.23 V_RHE_ (Figure 15a). The IPCE in both cases peaked at 674 nm, reaching 82 % at 1.23 V_RHE_, and 11 % at 0.8 V_RHE_, showing significant electron‐hole recombination occurring without the higher bias potential. At both voltages, a dip in IPCE to 0 % was observed at around 580 nm, corresponding to the wavelength of yellow light matching the colour of the photoanode (Figure 15b).

**Figure 16:**
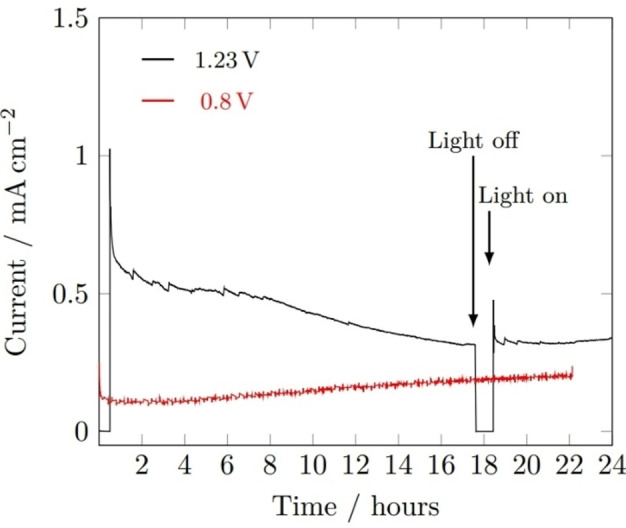
Photocurrent‐time curves of BiVO_4_ film at applied bias of 1.23 V_RHE_ (black) and 0.8 V_RHE_ (red) under 1 sun illumination (AM 1.5 G, 100 mW cm^−2^). Measurements performed in 1 M buffered potassium phosphate (pH 6.6).

Chronoamperometry stability tests carried out at applied voltages of 1.23 V_RHE_ and 0.8 V_RHE_ (Figure 16) revealed the instability of BiVO_4_ at 1.23 V_RHE_, possessing as a steady drop in photocurrent density from 1 hour onwards, overall decreasing from 0.6 mA cm^−2^ to approx. 0.3 mA cm^−2^. Comparatively, there were no signs of instability in the sample held at 0.8 V_RHE_, after the initial sharp drop associated with equilibration of the photoelectrochemical cell. The steady rise in photocurrent observed throughout the 0.8 V_RHE_ experiment, and in the last 2 hours of the 1.23 V_RHE_ experiment, can be linked with the heating of the photoelectrochemical cell due to prolonged exposure to simulated sunlight, increasing the surface reaction rates.

While the percentage decrease in performance due to BiVO_4_ corrosion was only significant at 1.23 V_RHE_, the stable photocurrent measured between 19–24 hours was still greater than that measured at 0.8 V_RHE_. It is likely that all surface V had dissolved (reported to be the most significant contributor to BiVO_4_ corrosion in phosphate‐based electrolytes)^[49]^ leaving behind a Bi_2_O_3_‐rich surface that was less susceptible to dissolution. This instability exemplifies the importance of coating BiVO_4_ with a protective layer, either a complementary semiconductor (e. g. Fe_2_O_3_) for increased visible light absorption and more efficient charge separation, or an electrocatalyst to improve the surface charge transfer to electrolyte, or both.

### Tungsten‐Doped BiVO_4_

The far superior performance of the BiVO_4_ films under rear‐side illumination suggests poor charge carrier conductivity, hence an analogous WO_3_ precursor (compound **5**) was developed and used to deposit W‐doped BiVO_4_ (W‐BiVO_4_) to improve overall device performance.[^17^, ^50^]

Doping of BiVO_4_ with tungsten, in which V^5+^ ions are replaced by W^6+^ ions,^[14]^ can enhance carrier density and create lattice defects resulting from both the different in electronic configurations between V and W, as well as the difference in the atomic sizes between the two atoms, thus enhancing BiVO_4_’s n‐type properties and has several positive effects: i. e., improved photocurrent, improved charge separation, improved surface charge transfer efficiency, lower surface charge transfer resistance and an enhanced visible light response.,[^33^, ^51^, ^52^]

By keeping the ligand system of the precursors consistent, integration of the WO_3_ precursor into the deposition process was possible without changing deposition parameters such as deposition temperature and solvent. A deposition study of just WO_3_ was first conducted to identify its properties before adding it to the BiVO_4_ process, data and discussion for which can be found in section S2 (ESI).

The deposition conditions for W‐BiVO_4_ were identical to BiVO_4_, briefly, a 40 min cold‐wall process utilising a total 30 mM precursor solution of Bi and V oxide precursors (15 mM of each). The molar percentage of the added tungsten precursor was subtracted from the percentage of V precursor in the solution, with W‐dopant concentrations of between 1–10 % used. W was confirmed to be present in the BiVO_4_ film by XRD, Raman, and EDX, and UV/Vis spectroscopy showed a very minor widening of the bandgap, all of the data for which can be found in section S3 (ESI). X‐ray diffraction data and Raman spectra of the W‐BiVO_4_ thin films (Figures S6 and S7, ESI) agree well with data of BiVO_4_ (see PDF#14‐0688) with only minor changes observed for the W‐BiVO_4_ film compared to pristine BiVO_4_, and no peaks belonging to other phases are present, except those from the substrate FTO (SnO_2_). These results demonstrate that W doping causes no obvious changes in the morphology and crystallinity of BiVO_4_ an observation which has been made previously for systems with between 0.58–1.8 % Wt% W incorporation.^[17]^ EDX measurements of the W‐BiVO_4_ films were performed (shown in ESI, Figure S8), with spectra showing Bi, V and O in similar ratios to the undoped films *vide supra*. The measured amounts of tungsten in the films grown using a tungsten‐doped precursor solution were between 0.02 At.% (1 mol % of compound **5**) 1.2 At.% (3 mol % of compound **5**) 1.6 At.% (5 mol % of compound **5**) and 2.0 At.% (10 mol % of compound **5**), as determined by EDX. Notably the measured values of tungsten quantity in the film were significantly lower than that of the precursor solution.

**Figure 17:**
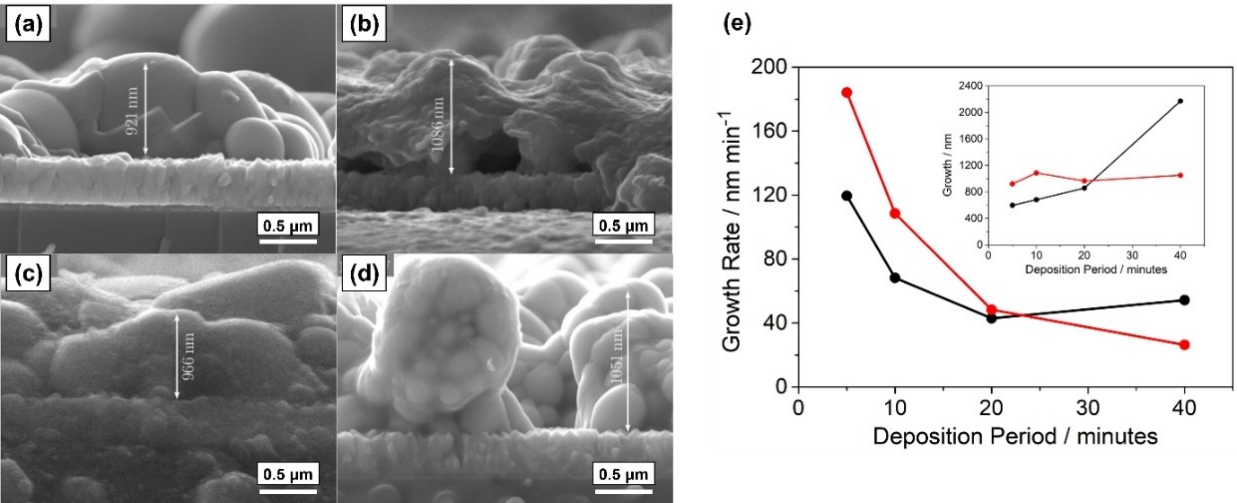
Cross‐sectional electron micrographs of (a) 1 %, (b) 3 %, (c) 5 %, (d) 10 % W‐doped BiVO_4_ films deposited using compounds **2**, **3** and **5** and a deposition time of 40 min. (e) Comparison of growth rate to the deposition period for BiVO_4_ (black) and W‐BiVO_4_ (red) thin films, inset shows the total growth for the same films over the deposition period.

The morphology of the W‐BiVO_4_ films were similar to the undoped films (Figure 17a–d), with a porous pillar‐like structure. The porosity of the material was reduced compared to the undoped BiVO_4_, with each ‘pillar’ being wider and more stunted. Overall, the thickness of all the W‐BiVO_4_ films were constant and approximately 1 μm, half the thickness of the pristine BiVO_4_ synthesised over the 40 min deposition time. The presence of compound **5** therefore had an inhibitive effect on the deposition rate of the Bi and V precursors, as well as itself being slower to deposit. Increasing the concentration of **5** (from 1–10 %) showed no further decreased in growth rate, hence we surmise that the inhibitive effect is a result of the presence of **5** only, and not the relative amount. This notable and unusual impact resulting from the inclusion of the WO_3_ precursor on the deposition rate and deposition saturation is interesting and will be subject to further study.

Analysis of the W‐BiVO_4_ thin films produced from precursor solutions containing 1 mol %, 3 mol %,5 mol % and 10 mol % of **5**, shown no discernible changes in the PXRD spectra of these films compared to as‐deposited BiVO_4_. These observations are consistent with those made by others in which loadings up to 9 At.% of W have no discernible impact on the crystallinity/morphology or phase separation of the composite materials. Tungsten loadings greater than 9 % At.% have been shown to result in distortion of the BiVO4 structure.^[33]^

**Figure 18:**
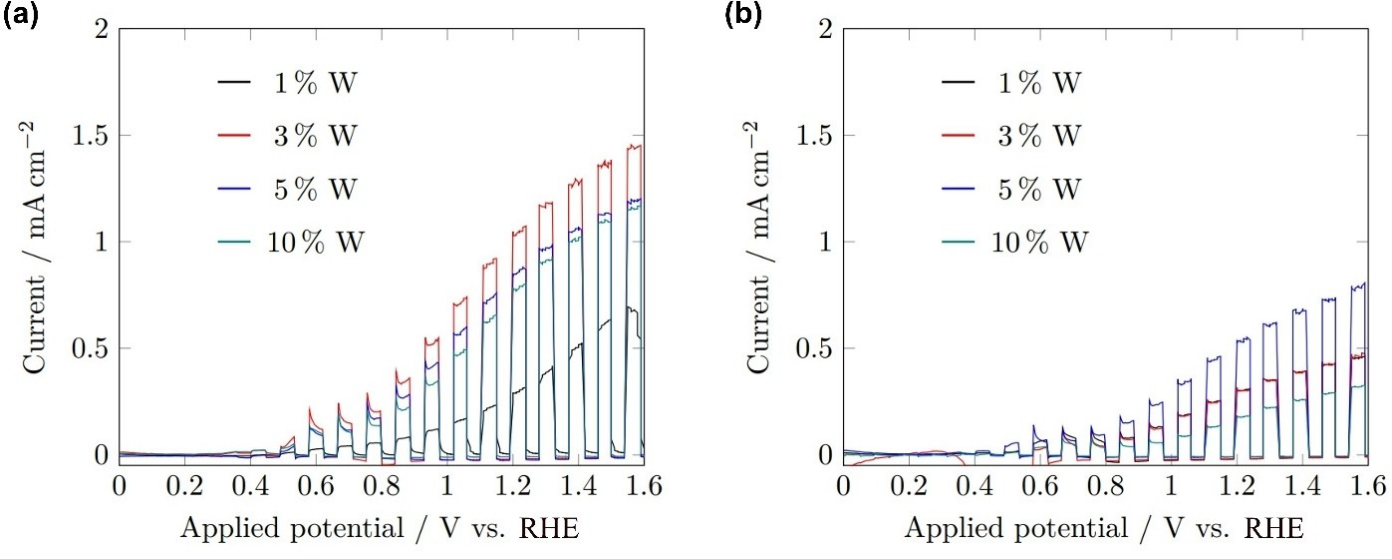
Linear sweep voltammograms of W‐BiVO_4_ films deposited for 40 min under 1 sun chopped (5 s on/off) (a) front‐side, (b) rear‐side illumination (AM 1.5 G, 100 mW cm^−2^) with molar percentage of W precursor of 1 % (black), 3 % (red), 5 % (blue) and 10 % (teal). All measurements performed in 1 M buffered potassium phosphate electrolyte (pH 6.6) with a scan rate of 10 mV s^−1^.

In contrast to the undoped films, the photocurrents generated by the W‐BiVO_4_ films were superior when illuminated from the front compared to the rear (Figure 18), with the highest photocurrent densities for each being around 1.1 (3 % W in precursor mix) and 0.6 mA cm^−2^ (5 % W in precursor mix), respectively, at an applied voltage of 1.23 V_RHE_. However, compared to the undoped films, the best photocurrent density produced by the W‐BiVO_4_ was lower, attributed to the reduction in thickness (inferior light absorption) and surface area (fewer catalytically active sites) as a result of the slower growth rate. Comparing the 40‐minute deposited W‐BiVO_4_ with 20‐minute deposited BiVO_4_ (films of more similar thickness, 1086 nm and 858 nm, respectively), photocurrents from rear‐side illumination are still greater than front for BiVO_4_ and lower for W‐BiVO_4_, hence the switching with W‐doping must be a result of improved charge transfer and not simply reduced thickness. Unfortunately, it was not possible at this point in time to run a deposition over a longer time period to produce W‐BiVO_4_ film of comparable thickness to the BiVO_4_ film at 40 minutes, however, studies on the effect of differing deposition times and thicknesses of the doped films are ongoing. The maximum photocurrent at 1.23 V_RHE_ observed for W‐BiVO_4_ (1.10 mA cm^−2^) is now also greater than that for BiVO_4_ (0.44 mA cm^−2^). Comparison of the best photocurrents observed in BiVO_4_ and W‐BiVO_4_ for both front‐ and rear‐side illumination are presented in Table 1.

**Table 1:** Comparison of the highest achieved photocurrent densities of BiVO_4_ and W‐BiVO_4_ films from both front and back illumination at 1.23 V_RHE_. Both W‐BiVO_4_ films were deposited for 40 minutes.

Irradiation Direction	j (BiVO_4_)/mA cm^−2^	j (W‐BiVO_4_)/mA cm^−2^
Front‐illumination	0.43 (5 min deposition)	1.10 (3 % W)
Rear‐illumination	1.23 (40 min deposition)	0.60 (5 % W)

**Figure 19:**
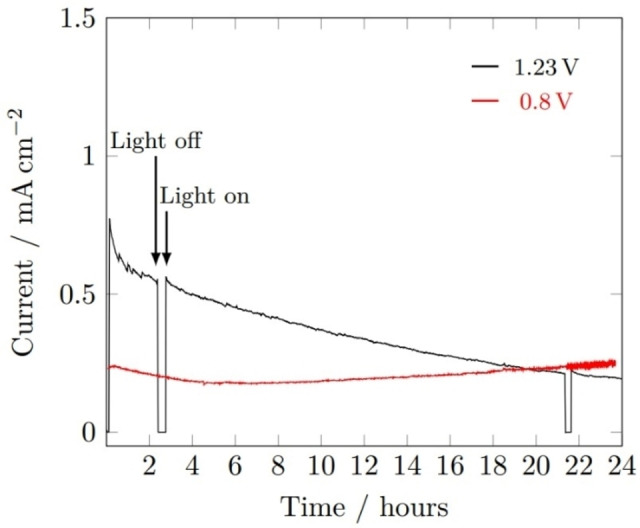
Photocurrent‐time curves of W‐BiVO_4_ film at applied bias of 1.23 V_RHE_ (black) and 0.8 V_RHE_ (red) under 1 sun illumination (AM 1.5 G, 100 mWcm^−2^). Measurements performed in 1 M buffered potassium phosphate (pH 6.6).

Stability tests were carried out analogously to those for undoped BiVO_4_ (Figure 19), showing mostly similar trends for both voltages. The only significant difference for the W‐BiVO_4_ film at 1.23 V_RHE_ was a non‐stable, still decreasing performance after 24 hours. W‐doping has not been reported to reduce the stability of BiVO_4_, however WO_3_ is thermodynamically unstable in pH 6.6 electrolyte at both 0.8 and 1.23 V_RHE_, hence the instability could be due to the presence of isolated phases of WO_3_ in the BiVO_4_ film, especially considering the 0.8 V_RHE_ measurement also had a photocurrent decrease over the first 5 hours instead of zero decrease for undoped.

**Figure 20:**
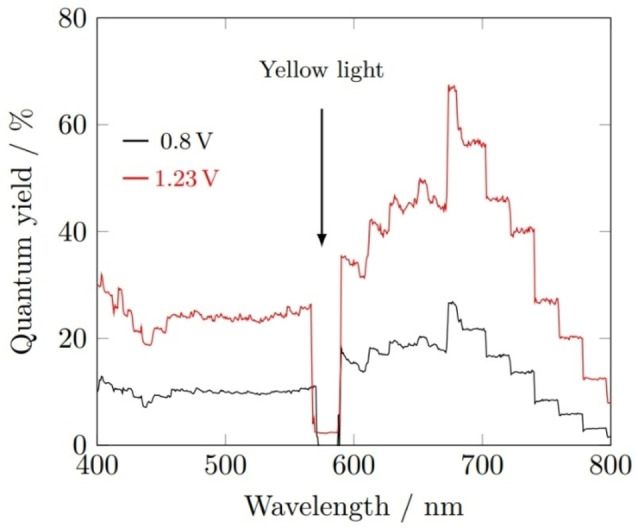
IPCE spectra for W‐doped BiVO_4_ films at 0.8 V_RHE_ (black) and 1.23 V_RHE_ (red). Measurements performed in 1 M buffered potassium phosphate (pH 6.6).

IPCE measurements were conducted on 3 % W‐doped BiVO_4_ under front‐side illumination (Figure 20). At an applied voltage of 1.23 V_RHE_, the conversion efficiency was approximately 10 % lower than that of the undoped films but was over double at 0.8 V_RHE_. There was therefore a reduced frequency of electron‐hole recombination in the W‐BiVO_4_ samples, and hence the electron mobility was higher, contributing towards the significant improvement in front‐side illumination photocurrent density.

**Figure 21:**
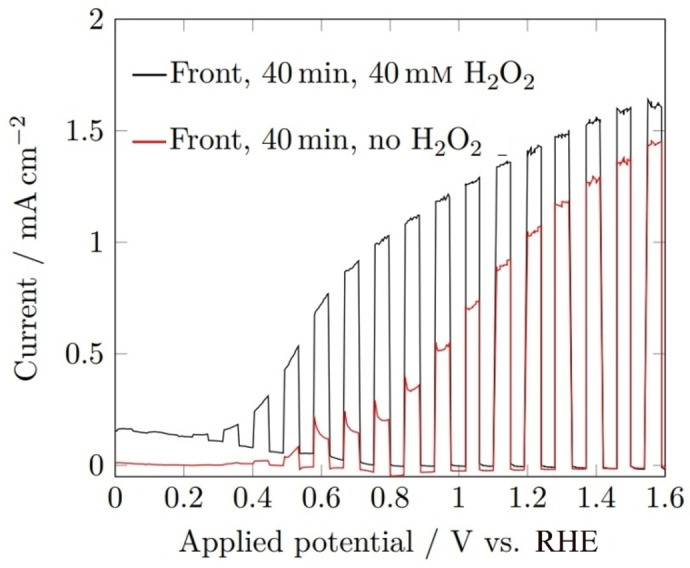
Linear sweep voltammograms of W‐BiVO_4_ films deposited for 40 min under 1 sun chopped (5 s on/off) rear illumination (AM 1.5 G, 100 mWcm^−2^), performed in 1 M buffered potassium phosphate electrolyte (pH 6.6) with 40 mM H_2_O_2_ (black), no H_2_O_2_ (red); using a scan rate of 10 mV s^−1^.

PEC measurements conducted under simulated sunlight in the presence of a hole acceptor (H_2_O_2_) (Figure 21) indicated that hole transfer to the electrolyte was still limited under normal operating conditions. While H_2_O_2_ is considered a poor “hole‐scavenger” due to its complicated decomposition pathways and interaction with the semiconductor surface, its use provides an upper limit for the bulk‐limited photocurrents.^[53]^

At 1.23 V_RHE_, the difference between the photocurrent density with and without the hole acceptor was significantly smaller than the undoped BiVO_4_ (0.25 vs 0.74 respectively), however the total current with the acceptor was drastically lower, implying that the peak performance of the device had decreased because of doping. As discussed previously, this latter observation is a result of the thinner W‐BiVO_4_ film. Work is in progress to further understand the influence of the W precursor on the deposition process, as well as optimising the W‐BiVO_4_ thin films to obtain more comparable photoanodes. It is also obvious that both doped and undoped films will benefit from the layering of a suitable co‐catalyst and/or passivating layer to promote charge separation/reduce recombination and increase stability.

## Conclusions

New, bespoke, and compatible precursors for the deposition of bismuth(III) oxide and vanadium(V) oxide were developed using tripodal amino alcohol ligands and characterised by NMR, single X‐ray diffraction, elemental analysis and thermogravimetric analysis. Phase‐pure monoclinic BiVO_4_ was deposited with a high degree of control over stoichiometry due to the high compatibility (solubility and thermal decomposition pathways) of the precursors. The resulting films were characterised by XRD, Raman spectroscopy, EDX, FE‐SEM, and UV‐visible spectroscopy. The optimal deposition temperature was found to be 400 °C, the lowest temperature of any previously reported AACVD‐grown BiVO_4_. Deposition time could not be fully optimised due to limitations with the setup, with the longest trialled duration of 40 minutes showing the best water splitting performance.

The BiVO_4_ nanostructured films produced appreciable photocurrents in the photoelectrochemical water‐splitting reaction, with a maximum value of 1.23 mA cm^−2^ under back‐side illumination at 1.23 V_RHE_ in buffered potassium phosphate electrolyte (pH 6.6), the largest photocurrent observed for pristine BiVO_4_ grown by CVD method to date. Front‐side illumination photocurrent densities were significantly lower due to poor electron transport from the interface to the back contact, which is a known issue in BiVO_4_ films. An impressive peak IPCE value of 82 % for 674 nm light at 1.23 V_RHE_ was recorded. The strengths of the AACVD technique combined with bespoke molecular precursor design was exemplified by the integration of a compatible WO_3_ precursor, which resulted in the deposition of W‐doped BiVO_4_ thin films with high control over dopant concentrations. While the At% of Bi : V :W are as yet undetermined, it is clear that co‐deposition of **2**, **3** and **5** leads to an enhanced PEC response. As part of a continuing investigation, we hope that further investigation of the W‐doped BiVO_3_ systems by XPS and varying the W concentration will reveal the limits of the doping effect. Nevertheless, it is clear we see no evidence of phase separation and formation of WO_3_ in the thin films, with the W dopant yielding films with greater electron‐hole separation and charge transfer properties, however overall film growth rates were significantly reduced. Work is ongoing to understand the impact of the W‐precursor inclusion into the deposition process and film growth mechanisms.

## Experimental

NMR experiments were conducted at 298 K, on a Bruker AV300 spectrometer which was operated at 300.22 MHz (1H) and 75.49 MHz (^13^C{^1^H}) or a 500 MHz Agilent Propulse spectrometer which was operated at 500.13 MHz (^1^H) and 125.76 MHz (^13^C{^1^H}). NMR samples of air‐sensitive compounds were made up in Young's tap NMR tubes, prepared in a glove box under an argon atmosphere. TGA was carried out on a SETSYS Evolution machine using CALISTO data acquisition software, and all TGA experiments were performed under a blanket of argon (initial 30 min purge at room temperature) with a temperature ramp of 5 °C min^−1^.

All crystallographic data was collected at 150(2) K on a SuperNova, Dual, EosS2 diffractometer using radiation Cu−Kα (*λ*=1.54184 Å) or Mo−Kα (*λ*=0.71073 Å). All structures were solved by direct methods followed by full‐matrix least squares refinement on *F* ^*2*^ using the WINGX‐2014 suite of program^[54]^ or OLEX2.^[55]^ All hydrogen atoms were included in idealised positions and refined using the riding model. Crystals were isolated from an argon filled Schlenk flask and immersed under oil before being mounted onto the diffractometer. All structural data was obtained and refined by Dr Andrew Johnson.

Powder X‐ray diffraction (PXRD) experiments were run on a Bruker AXS D8 ADVANCE using DIFFRAC Plus XRD Commander software or a STOE STADI P using WinXPow software. Raman spectra were recorded on a Renishaw inVia system using either a 532 nm (green) or 325 nm (UV‐HeCd) laser at varied energy intensity and exposure times. Electron micrographs were obtained on a JEOL‐6301F or JEOL‐7900F spectrometer, and EDX analyses were performed on a JEOL JSM‐6480LV spectrometer with an INCA X‐Act silicon drift detector.

Photoelectrochemical measurements were obtained using an Ivium Technologies CompactStat. Potentiostat in a 3‐electrode setup using a platinum wire counter electrode and saturated Ag/AgCl reference electrode. Thin film sample working electrodes were illuminated by simulated sunlight (AM 1.5 G, 100 mW cm^−2^) from a filtered 300 W Lot Quantum Design xenon lamp source.

All reactions were performed under inert conditions, standard Schlenk line and glove box techniques under an Argon atmosphere. All solvents used in air and moisture sensitive reactions were dried using a commercially available solvent purification system (Innovative Technology Inc., MA), except for tetrahydrofuran (THF) and diethyl ether, which were dried over potassium in a still. Dry solvents also were degassed under argon prior to use. Deuterated NMR solvents were purchased from Fluorochem, UK. Deuterated solvents that were used for air and moisture sensitive products were dried over potassium before isolating via vacuum distillation. All dry solvents were stored under argon in Young's ampoules over 4 Å molecular sieves. Ammonia (4 % in methanol) was purchased from TCI chemicals, and all further reagents were purchased from Sigma‐Aldrich and were used as supplied.

Fluorine‐doped tin oxide (FTO) coated glass substrates were pre‐cleaned with soapy water and isopropyl alcohol, then dried under N_2_ flow and further cleaned using a low pressure O_2_ plasma reactor for 15 minutes. The substrate was then immediately placed into the quartz tube for AACVD.

Thin films were deposited using a cold‐wall rig coupled with a TSI 3076 Constant Output Atomiser. Argon was used as a carrier gas, monitored by a Bronkhorst flow meter set at a constant rate of 1.5 Lmin^−1^. Precursor solutions were made up in dry THF inside a glovebox.

The rig was purged with argon for 1 hour with the pot isolated and sealed with inert atmosphere, and the substrate heated. The precursor pot was then opened to the system, injecting the precursor as a fine aerosol. After the desired deposition time, the pot was closed, and the substrate allowed to cool to room temperature under argon flow. The films could then be removed from the system and, if required, annealed in a furnace at 540 °C for 2 hours in ambient air.

### Precursor Synthesis

#### [N{CH_2_Me_2_COH}_3_] (1)

Dimethyloxirane (16 g, 221.8 mmol) was added to a 2 M solution of ammonia in methanol (35 mL, 69.3 mmol) and heated to 50 °C for 96 h in a sealed reaction vessel. The resultant clear, colourless reaction mixture was concentrated in vacuo to give a clear, colourless oil. This oil was dissolved in hexanes, leaving an insoluble white solid from which the hexanes were decanted. The solid was further washed with hexanes until all trace of the dimethyloxirane was removed. Because of the notable hygroscopic nature of compound **2**, it was dissolved in dichloromethane and dried with magnesium sulphate (10 min) before being filtered into a schlenk flask, dried *in‐vacuo*, and stored under argon as a fine white powder (15.116 g, 93 %).

^1^H NMR (CDCl_3_, 500 MHz): 2.73 (d, 6H, NC ***H***_***2***_), 1.10 (s, 18H, C(C ***H***_***3***_)_2_).

^13^C^1^H NMR (CDCl_3_, 125.7 MHz): 71.15 (***C*** OH), 70.38 (***C*** OH), 70.09 (***C*** OH), 61.19 (***C*** H_2_N), 28.88 (***C*** H_3_), 27.56 (***C*** H_3_).

#### [(O)V{OCMe_2_CH_2_}_3_N] (2)

A solution of VO(O^*i*^Pr)_3_ (1.954 g, 8.0 mmol) in THF (10 mL) was added to a suspension of **1** (1.867 g, 8.0 mmol) in THF (20 mL). After stirring for 3 hrs THF was removed *in‐vacuo*. The yellow residue was dissolved in a minimum of dry toluene (6 ml), filtered through a celite plug, concentrated, and stored at −28 °C to yield pale yellow crystals. The crystals were isolated from the liquor, and the liquor subsequently concentrated to yield a second portions of crystals. The resulting crystals were dried under vacuum, yielding 1.434 g (60 %) of yellow crystals.

^1^H NMR (500 MHz, C_6_D_6_) δH: 1.09 (6H, s, NCH_2_C ***Me***_***2***_O), 2.45 (2H, s, NC ***H***_***2***_CMe_2_O).

^13^C^1^H NMR (125.7 MHz, C_6_D_6_) δC: 29.7 (NCH_2_C ***Me***_***2***_O), 70.6 (N ***CH***_***2***_CMe_2_O), 85.3 (NCH_2_ ***C*** Me_2_O).

^51^V NMR (131.4 MHz, C_6_D_6_) δV: −423.8. IR n_max_ (cm^−1^): 660, 799, 909, 934 (V=O), 1085, 1142, 1281, 1363, 1383, 1456, 2881, 2922, 2975.

Elemental Analysis: Found (Calculated) C: 48.48 (48.49) H: 8.23 (8.14) N: 4.895 (4.71).

#### [Bi{OCMe_2_CH_2_}_3_N] (3)

A solution of Bi{N(SiMe_3_)_2_}_3_ (5.5 g, 8.0 mmol) in THF (10 mL) was added to a suspension of **1** (1.867 g, 8.0 mmol) in THF (20 mL). A yellow solution immediately formed, the colour of which began to fade as the reaction proceeded. The reaction mixture was stirred for 12 hrs at room temperature resulting in a clear, colourless solution, which was concentrated *in‐vacuo* and redissolved in dry toluene and filtered through celite. Compound **3** was recrystallised from the solution as colourless blocks (2.32 g, 66 %).

^1^H NMR (C_6_D_6_, 500 MHz) δH: 1.29 (6H, s, NCH_2_C ***Me***_***2***_O), 2.63 (2H, s, NC ***H***_***2***_CMe_2_O). ^13^C^1^H NMR (C_6_D_6_, 125.7 MHz) δC: 33.8 (NCH_2_C ***Me***_***2***_O), 73.2 (N ***CH***_***2***_CMe_2_O), 75.1 (NCH_2_ ***C*** Me_2_O).

Elemental Analysis: Found (Calculated) C: 32.86 (32.81) H: 5.56 (5.51) N: 3.22 (3.19)

#### [W(N^t^Bu)(NH^t^Bu){(OCMe_2_CH_2_)_3_N}] (4)

A solution of **1** (1.867 g, 8.0 mmol) in dry THF (10 mL) was added slowly to a solution of W(N^t^Bu)_2_(NH^t^Bu)_2_ (3.76 g, 8 mmol) in dry THF (20 mL). Upon addition, the reaction mixture maintained a clear yellow appearance. After the reaction mixture was left to stir for 12 hrs, the solvent was removed *in‐vacuo* and entrenched THF was a removed by washing with dry hexanes. Trace hexanes were removed *in‐vacuo* with gentle heating to yield a yellow semi‐solid (3.26 g, 73 %).

^1^H NMR (C_6_D_6_, 500 MHz) δH: 1.37 (s, 9H, NC ***Me***_3_), 1.44 (s, 9H, NC ***Me***_3_), 1.51 (s, 6H, OCH_2_C ***Me***_2_), 1.59 (s, 6H, OCH_2_C ***Me***_2_), 1.78 (s, 6H, OCH_2_C ***Me***_2_), 2.60 (m, 4H, OC ***H***_***2***_CMe_2_), 2.78 (m, 4H, OC ***H***_***2***_CMe_2_), 3.29 (d, 4H, OC ***H***_***2***_CMe_2_) 8.07 (br s, 1H, NH). ^13^C^1^H NMR (C_6_D_6_, 125.7 MHz) δC: 29.84 (NCH_2_C ***Me***_2_), 31.2 (N(H)C ***Me***_3_), 31.8 (NCH_2_C ***Me***_2_), 32.1, (NC ***Me***_3_), 33.6 (NCH_2_C ***Me***_2_), 58.5 (N(H) ***C*** Me_3_), 71.5 (NCH_2_ ***C*** Me_2_O), 73.2 (NCH_2_ ***C*** Me_2_O), 73.5 (N ***C*** Me_3_), 73.8 (NCH_2_ ***C*** Me_2_O), 71.8 (N ***C*** H_2_CMe_2_O), 79.7 (N ***C*** H_2_CMe_2_O), 82.6 (N ***C*** H_2_CMe_2_O).

Elemental Analysis: Found (Calculated) C: 42.63 (43.09) H: 7.66 (7.78) N: 7.33 (7.54)

#### [(μ‐O){W(N^t^Bu)(OCMe_2_CH_2_)_3_N}_2_] (5)

Water (54 mL, 3.0 mmol) in THF (10 ml) was added over the course of 1 min to a solution of **4** (6 mmol), prepared as above, in THF (20 mL). The mixture was allowed to stand for 1 hr, whereupon the solvent was removed at reduced pressure. Hexane (5 mL) was added to aid in the removal of the last traces of THF. The residue was dissolved in 20 mL of hot toluene, which upon cooling afforded colourless crystals of 5 as the toluene solvate (2.85 g, 81 % based on W).

^1^H NMR (C_6_D_6_, 500 MHz) δH: 1.49 (s, 6H, OCH_2_C ***Me***_2_), 1.55 (s, 9H, NC ***Me***_3_), 1.76 (s, 12H, OCH_2_C ***Me***_2_), 2.98 (s, 4H, OC ***H***_***2***_CMe_2_), 3.79 (d, 4H, OC ***H***_***2***_CMe_2_). ^13^C^1^H NMR (C_6_D_6_, 125.7 MHz) δC: 30.14 (NCH_2_C ***Me***_2_), 31.94 (NCH_2_C ***Me***_2_), 32.42 (NC ***Me***_3_), 68.4 (NCH_2_ ***C*** Me_2_O), 69.2 (NCH_2_ ***C*** Me_2_O), 71.5 (NCH_2_ ***C*** Me_2_O), 72.3 (N ***C*** Me_3_), 81.84 (N ***C*** H_2_CMe_2_O), 86.70 (N ***C*** H_2_CMe_2_O).

Elemental Analysis: Found (Calculated) C: 46.66 (47.19) H: 7.04 (7.06) N: 4.85 (4.76).

### Crystallographic Data

CCDC 2351161, 2351163 and 2351162 contain the supplementary crystallographic data for this paper. These data can be obtained free of charge at www.ccdc.cam.ac.uk/conts/retrieving.html [or from the Cambridge Crystallographic Data Centre, 12, Union Road, Cambridge CB2 1EZ, UK; fax: + 44–1223/336‐033; E‐mail:deposit@ccdc.cam.ac.uk.

## Author Contributions

T. R. Harris‐Lee, M. K. Surman and A. L. Johnson prepared the original draft; M. K. Surman and A. J. Straiton carried out precursor synthesis and analysis; M. K. Surman performed all deposition experiments and photoelectrochemical characterisation; A. L. Johnson collected and processed all single crystal X‐ray data; F. Marken assisted M. K. Surman and T. R. Harris‐Lee in electrochemical analysis. All authors reviewed and edited the final document.

## Conflict of Interests

The authors declare no conflict of interest.

1

## Supporting information

As a service to our authors and readers, this journal provides supporting information supplied by the authors. Such materials are peer reviewed and may be re‐organized for online delivery, but are not copy‐edited or typeset. Technical support issues arising from supporting information (other than missing files) should be addressed to the authors.

Supporting Information

Supporting Information

Supporting Information

Supporting Information

## Data Availability

The data that support the findings of this study are available in the supplementary material of this article.
