# Supplementary material for: Precursor Development and Aerosol‐Assisted Chemical Vapour Deposition for BiVO4 and W‐Doped BiVO4 Photoanodes: A Universal Ligand Approach
Source: ChemSusChem. 2024 Nov 8;18(4):e202401452. doi: 10.1002/cssc.202401452 (PMC11826119; doi:10.1002/cssc.202401452)
Supplement: Supplementary file 4 — Supporting Information [file CSSC-18-e202401452-s002.pdf]

ChemSusChem

Supporting Information

# Precursor Development and Aerosol-Assisted Chemical Vapour Deposition for $BiVO_4$ and W-Doped $BiVO_4$ Photoanodes: A Universal Ligand Approach

Thom R. Harris-Lee, Matthew K. Surman, Andrew J. Straiton, Frank Marken, and Andrew L. Johnson*

**Electronic Supplementary Information:**

# Precursor Development and Aerosol-Assisted Chemical Vapour Deposition for $BiVO_4$ and W-doped $BiVO_4$ Photoanodes: A Universal Ligand Approach

*Thom R. Harris-Lee [a,b], Matthew K. Surman [a], Andrew J. Straiton [a], Frank Marken [a], Andrew L. Johnson* [a]*

[a] *Department of Chemistry, University of Bath, Claverton Down, Bath, BA2 7AY, UK*

[b] *School of Chemistry, Monash University, Clayton, Vic 3800, Australia*

## Table of Contents

S1. Thermal Characterisation ........ 2
Figure S1 ........ 2
Figure S2 ........ 3
Figure S3 ........ 3
Table S1 ........ 4
S2. $WO_3$ Deposition Study ........ 4
Figure S4 ........ 4
Figure S5 ........ 5
S3. W-$BiVO_4$ Film Characterisation ........ 5
Figure S6 ........ 5
Figure S7 ........ 6
Figure S8 ........ 6
Figure S9 ........ 7
S4. X-Ray Crystallography Data ........ 8
Table S2 ........ 8
Table S3 ........ 9
Table S4 ........ 10
References ........ 11

## S1. Thermal Characterisation

The thermogravimetric analysis trace for compound **2** contained two distinct steps, the transition between which is marked on Figure S1 with a dashed arrow. This suggests that the tridentate ligands were not removed from the complex intact, but rather were partially decomposed into product fragments before all three V-O bonds were broken. Following the initial mass loss temperature of 175 °C, a value of 43% was reached at a temperature of 230 °C, significantly greater than the 30.6% target for $V_2O_5$ formation. However, continued heating yielded steady mass loss to a final weight of 29.0%, close to the target, hence it is likely that $V_2O_5$ was formed at the 43% point, however residual caron deposits from ligand decomposition were present, which underwent pyrolysis under continued and increased heating.

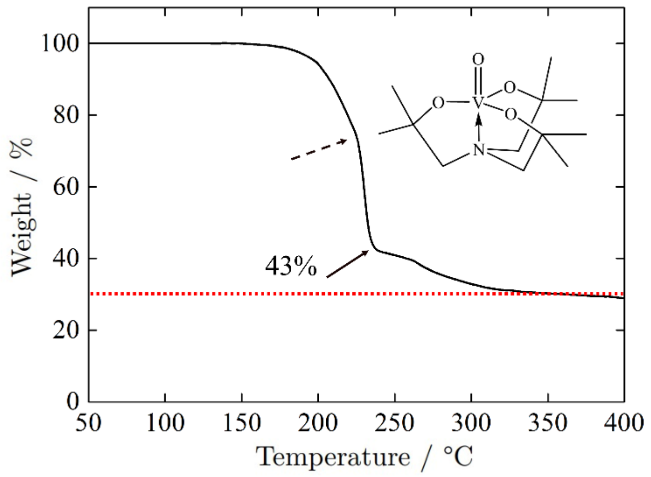


Figure S1. Thermogravimetric analysis of compound **2** measured in an inert Ar glovebox atmosphere between 30 and 520 °C at a constant ramp rate of 5 °C $min^{-1}$.

Thermogravimetric analysis showed that compound **3** (Figure S2) underwent initial mass loss at 163.2 °C after which point a single, smooth mass loss process occurred, finishing at 1%. **3** was therefore highly volatile, with no evidence of Bi or $Bi_2O_3$ formation (Table S1). The development of an extremely volatile precursor such as this provides more opportunity about how the precursor can be used; for example, **3** could be an excellent candidate for atomic layer deposition or low-pressure CVD of $Bi_2O_3$, in addition to the aerosol-assisted CVD methods targeted in these works.

Unfortunately, the TGA trace of a volatile precursor also cannot provide any detailed information regarding the decomposition of the precursor, only that the decomposition temperature is above 250 °C, the temperature at which volatilisation was complete.

Because a volatile precursor will interact with a heated substrate in a different manner to that of a non-volatile precursor, it is important to take these things into consideration when establishing a deposition process involving a precursor solution containing two unique precursors. For example, with a precursor solution containing V-source compound **2** and Bi-source compound **3**, any given increase in substrate temperature may increase V incorporation through accelerated decomposition of **2**, but reduce incorporation of Bi through heightened repulsion of the volatile compound **3** - and *vice versa* for a reduction in substrate temperature. Therefore, when developing an AACVD procedure for $BiVO_4$ using these precursors, it was important to thoroughly investigate the effects of temperature on the relative amounts of Bi and V incorporated into the resultant film.

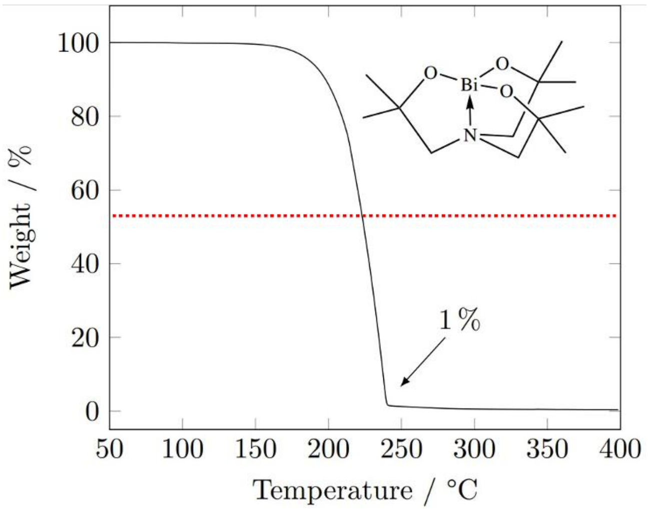


Figure S2. Thermogravimetric analysis of compound **3**, measured in an inert Ar glovebox atmosphere between 30 and 520 °C at a constant ramp rate of 5 °C $min^{-1}$.

The TGA of compound **5** (Figure S3), which was conducted under an atmosphere of argon, showed initial mass loss at around 141 °C, which could not be attributed to any expected decomposition processes in **5**. It was most likely that this was due to a small amount of residual ligand present in the sample, which may have dropped out of solution during the isolation of the crystals. The major mass-loss step that occurred at c.a. 240 °C likely corresponds to the decomposition of the bulk of **5** into $WO_3$, ending at 60 wt%. As highlighted in Table S1, the initial 'end' point (shown by an arrow in Figure S3) of the major decomposition step had a wt% value roughly 13% higher than the calculated value for $WO_3$. Between the temperatures of 240–400 °C this excess mass was steadily lost resulting in a final value of 50.4 wt%, which correlated with the expected value and suggested that the excess mass was trapped carbon-rich material due to mass transfer limitations of the decomposed ligand fragments. Despite the inability to substitute the tert-butylimido ligands on **5**, the TGA trace of this compound showed compatibility with compounds **2** and **3**, with the major decomposition steps all predominantly taking place within a similar temperature range.

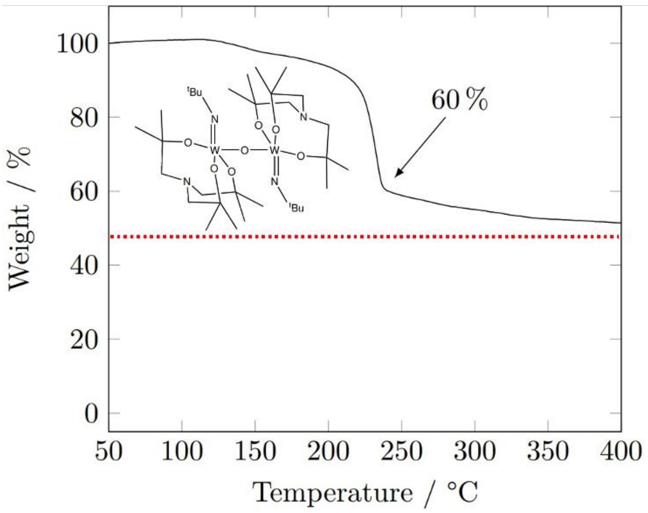


Figure S3. Thermogravimetric analysis trace of compound **5**, measured in an inert Ar glovebox atmosphere between 30 and 520 °C at a constant ramp rate of 5 °C $min^{-1}$.

Table S1**.** Thermogravimetric analysis data for vanadium (compound **2**), bismuth (compound **3**), and tungsten (compound **5**) precursors.

| Sample Identity | Target final weight / % | Observed final weight / % | Temperature of initial mass loss* / °C |
|---|---|---|---|
| Compound **2** | 30.6 | 29.0 | 175.1 |
| Compound **3** | 53.0 | 0.4 | 163.2 |
| Compound **5** | 47.0 | 50.4 | 141.0 |

*The temperature at which 1% mass loss has occurred.

## S2. $WO_3$ Deposition Study

To confirm deposition of $WO_3$ by AACVD of compound **5** under conditions identical to the optimised $BiVO_4$ growth condition, a 30 mM solution of **5** in dry tetrahydrofuran was deposited using a cold-wall AACVD process at 400 °C for 30 min. Whilst the TGA trace of **5** was similar to that of the Bi and V precursors, it appeared to deposit at a significantly lower rate, as after 30 min of deposition there was insubstantial material present to be detected by XRD. However, the presence of $WO_3$ in the post annealed (540 °C, 2 h) films was confirmed by FE-SEM and PEC experiments.

Top-down and cross-sectional FE-SEM images (Figure S4) of the films deposited using **5** clearly show that material had been deposited on top of the FTO: small, irregular shaped nanoparticles were visible from the top-down view, though the cross-sectional image showed the layer of this material was thin, appearing as only a light 'dusting' of the FTO surface.

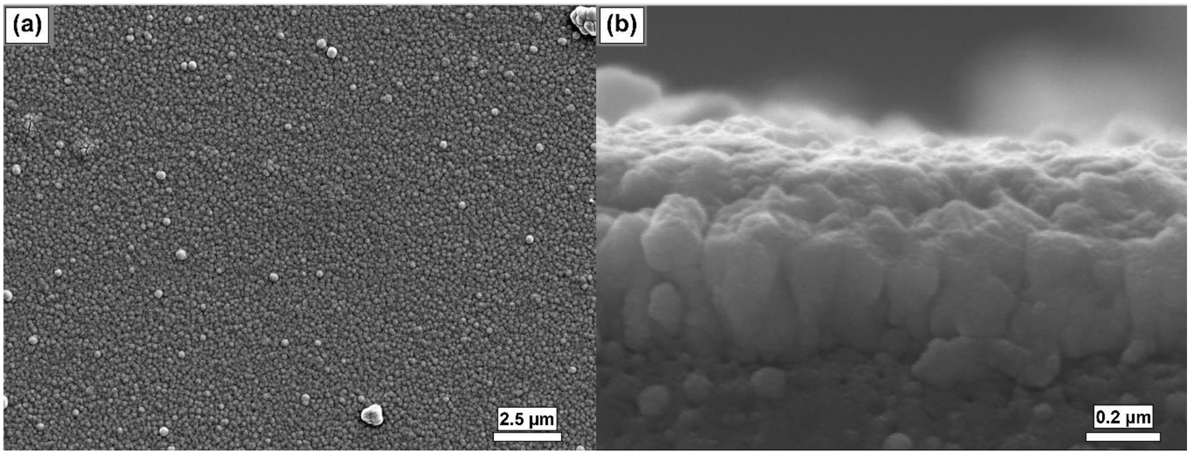


Figure S4. Electron micrographs of $WO_3$ thin films deposited using compound **5,** (a) top-down, (b) cross section.

PEC measurements of the film did not produce any measurable photocurrent, as expected due to the low thickness of the film. However, after PEC measurements there was an electrochromic change of the material from clear-colourless to clear-blue, attributed to reductive hydrogen intercalation in $WO_3$ films (Figure S5, insert).[1] Further, cyclic voltammetry measurements (Figure S5) showed a characteristic broad peak around 0.2–0.3 $V_{RHE}$ during the positive sweep, which corresponded to de-intercalation of the aforementioned protons.[2] Compound **5** could therefore be used to successfully deposit $WO_3$ films by the same AACVD process as the optimised $BiVO_4$ process, although seemingly at a much slower rate than the Bi and V precursors.

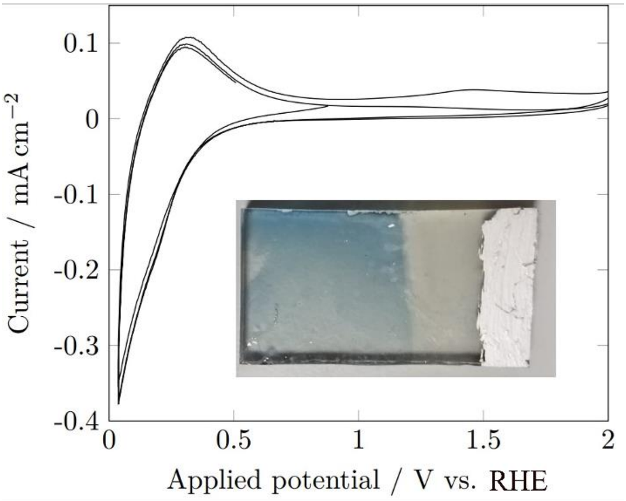


Figure S5. Cyclic voltammogram of the $WO_3$ film deposited using compound **5**, performed in 1 M buffered potassium phosphate electrolyte (pH 6.6) with a scan rate of 50 mV $s^{-1}$; insert: photograph of the film after the electrochromic effect of reduction had generated blue colour (insert).

## S3. W-$BiVO_4$ Film Characterisation

The XRD patterns were not significantly changed by the inclusion of the W dopant in the precursor solution, and still corresponded to the monoclinic scheelite crystal phase of $BiVO_4$ (Figure S6). There were minor changes to some of the XRD peaks corresponding to $BiVO_4$ such as peak intensity, broadness and splitting, which typically has been indicative of tungsten entering the $BiVO_4$ lattice, rather than the formation of two separate $WO_3$ and $BiVO_4$ phases.[3] However, if there was a separate $WO_3$ phase present, it would not be seen in the XRD pattern as the $WO_3$ films fabricated in the previous section were not thick enough to produce an XRD signal.

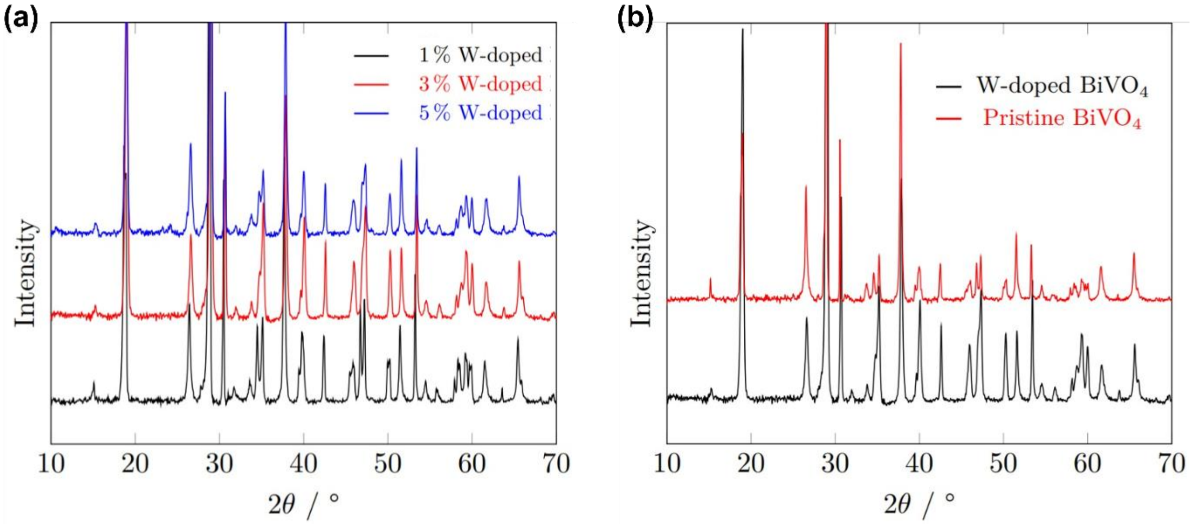


Figure S6. XRD patterns of (a) W-doped $BiVO_4$ films with W values between 1-5%, (b) undoped and 3% W-doped $BiVO_4$.

Raman spectra of the W-doped $BiVO_4$ films (Figure S7) were also very similar to those of the pristine $BiVO_4$ films, with no additional peaks present nor significant peak shifts. These findings were very

similar to those reported in the literature in which doping of $BiVO_4$ with W has not often caused significant changes in the Raman spectrum.[4,5] The most significant change is typically a slight shifting of the peak located at around 828 $cm^{-1}$, which corresponds to a $\nu_1$ stretching mode of $VO_4$ in monoclinic $BiVO_4$. Some minor shifting of this peak of approx. 10 $cm^{-1}$ was observed with higher doping concentrations of tungsten, corresponding to a distortion of the $VO_4$ units by the larger W ions.

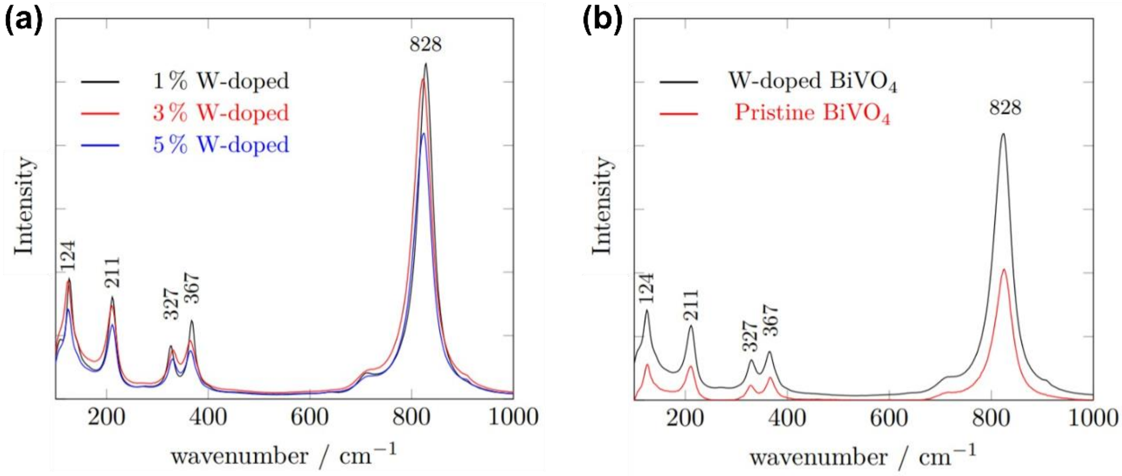


Figure S7. Raman spectra of (a) W-doped $BiVO_4$ films with W values between 1-5%, (b) undoped and W-doped $BiVO_4$.

EDX measurements of the W-doped $BiVO_4$ films (Figure S8) showed a similar ratio of Bi, V, and O to the undoped films. The measured amounts of W in the film grown using a 3% W-doped precursor solution was 1.2%. Notably, the measured values of W quantity in the film were significantly lower than that of the precursor solution, as expected given that compound **5** was found to deposit $WO_3$ at a significantly lower rate than **2** and **3** deposited $BiVO_4$.

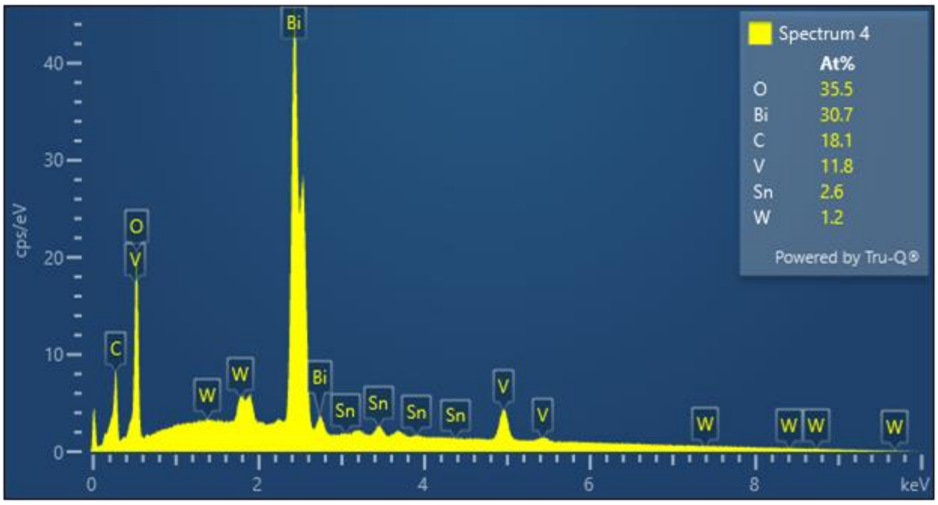


Figure S8. Energy-dispersive X-ray spectroscopy of post-annealed 3% W-doped $BiVO_4$ film.

UV-visible spectroscopy was used to generate a Tauc plot of the 3% W-doped $BiVO_4$ and estimate the band gap (Figure S9). The band gap values, 2.44 and 2.82 eV for indirect and direct band gaps respectively, were marginally wider than those found in undoped films (2.34 and 2.79), as expected given most literature reports no significant energy change because of W-doping of $BiVO_4$.[3,6]

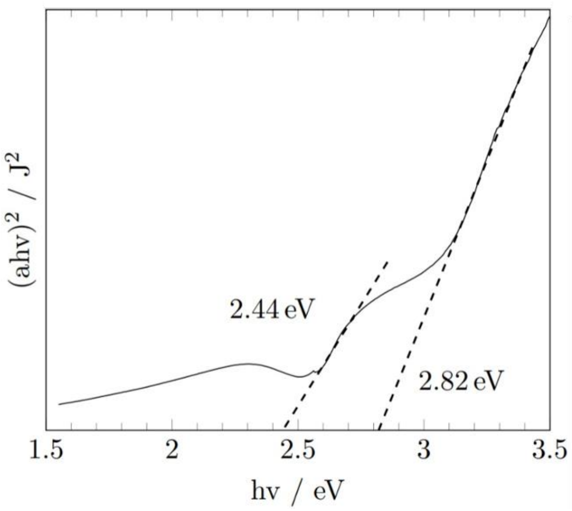


Figure S9. Tauc plot of 3% W-doped $BiVO_4$ film deposited using compounds **2**, **3**, and **5**.

# S4. X-Ray Crystallography Data

Table S2. Crystal data and structure refinement for compound **2**.

| | | |
|---|---|---|
| Identification code | **2** | |
| Empirical formula | C12 H24 N O4 V | |
| Formula weight | 297.26 | |
| Temperature | 150.00(10) K | |
| Wavelength | 1.54184 Å | |
| Crystal system | Hexagonal | |
| Space group | P63 | |
| Unit cell dimensions | a = 10.2734(3) Å | α = 90°. |
| | b = 10.2734(3) Å | β = 90°. |
| | c = 7.8637(2) Å | γ = 120°. |
| Volume | 718.76(5) $Å^3$ | |
| Z | 2 | |
| Density (calculated) | 1.374 $Mg/m^3$ | |
| Absorption coefficient | 5.839 $mm^{-1}$ | |
| F(000) | 316 | |
| Crystal size | 0.290 x 0.045 x 0.018 $mm^3$ | |
| Theta range for data collection | 4.971 to 72.452°. | |
| Index ranges | -12<=h<=12, -12<=k<=12, -9<=l<=6 | |
| Reflections collected | 4427 | |
| Independent reflections | 823 [R(int) = 0.0406] | |
| Completeness to theta = 67.684° | 100.0% | |
| Absorption correction | Gaussian | |
| Max. and min. transmission | 1.000 and 0.482 | |
| Refinement method | Full-matrix least-squares on F2 | |
| Data / restraints / parameters | 823 / 1 / 57 | |
| Goodness-of-fit on $F^2$ | 1.176 | |
| Final R indices [I>2sigma(I)] | R1 = 0.0249, wR2 = 0.0665 | |
| R indices (all data) | R1 = 0.0251, wR2 = 0.0666 | |
| Absolute structure parameter | 0.008(7) | |
| Extinction coefficient | n/a | |
| Largest diff. peak and hole | 0.203 and -0.214 $e.Å^{-3}$ | |
| CCDC Number | 2351161 | |

Table S3. Crystal data and structure refinement for compound **3**.

| | | |
|---|---|---|
| Identification code | **3** | |
| Empirical formula | C24 H48 Bi2 N2 O6 | |
| Formula weight | 878.60 | |
| Temperature | 150.01(10) K | |
| Wavelength | 0.71073 Å | |
| Crystal system | Monoclinic | |
| Space group | P21/n | |
| Unit cell dimensions | a = 9.4028(3) Å | α = 90°. |
| | b = 9.8718(4) Å | β = 90.956(3)°. |
| | c = 15.4969(5) Å | γ = 90°. |
| Volume | 1438.26(9) $Å^3$ | |
| Z | 2 | |
| Density (calculated) | 2.029 $Mg/m^3$ | |
| Absorption coefficient | 12.256 $mm^{-1}$ | |
| F(000) | 840 | |
| Crystal size | 0.573 x 0.453 x 0.287 $mm^3$ | |
| Theta range for data collection | 3.961 to 29.298°. | |
| Index ranges | -12<=h<=11, -13<=k<=11, -21<=l<=20 | |
| Reflections collected | 11787 | |
| Independent reflections | 3476 [R(int) = 0.0319] | |
| Completeness to theta = 25.242° | 99.7% | |
| Absorption correction | Analytical | |
| Max. and min. transmission | 0.066 and 0.015 | |
| Refinement method | Full-matrix least-squares on F2 | |
| Data / restraints / parameters | 3476 / 0 / 160 | |
| Goodness-of-fit on F2 | 1.108 | |
| Final R indices [I>2sigma(I)] | R1 = 0.0236, wR2 = 0.0517 | |
| R indices (all data) | R1 = 0.0299, wR2 = 0.0537 | |
| Extinction coefficient | n/a | |
| Largest diff. peak and hole | 0.693 and -1.831 $e.Å^{-3}$ | |
| CCDC Number | 2351163 | |

Table S4. Crystal data and structure refinement for compound **5**.

| | | |
|---|---|---|
| Identification code | **5** | |
| Empirical formula | C46 H82 N4 O7 W2 | |
| Formula weight | 1170.85 | |
| Temperature | 150.00(10) K | |
| Wavelength | 0.71073 Å | |
| Crystal system | Triclinic | |
| Space group | P-1 | |
| Unit cell dimensions | a = 11.1967(4) Å | α = 102.645(3)°. |
| | b = 12.9816(5) Å | β = 93.468(3)°. |
| | c = 17.1459(6) Å | γ = 90.044(3)°. |
| Volume | 2427.02(16) $Å^3$ | |
| Z | 2 | |
| Density (calculated) | 1.602 $Mg/m^3$ | |
| Absorption coefficient | 4.787 $mm^{-1}$ | |
| F(000) | 1180 | |
| Crystal size | 0.538 x 0.344 x 0.092 $mm^3$ | |
| Theta range for data collection | 3.586 to 27.580°. | |
| Index ranges | -14<=h<=14, -16<=k<=16, -22<=l<=22 | |
| Reflections collected | 18229 | |
| Independent reflections | 18229 [R(int) = ?] | |
| Completeness to theta = 25.242° | 99.7% | |
| Absorption correction | Analytical | |
| Max. and min. transmission | 0.669 and 0.179 | |
| Refinement method | Full-matrix least-squares on F2 | |
| Data / restraints / parameters | 18229 / 0 / 553 | |
| Goodness-of-fit on F2 | 0.944 | |
| Final R indices [I>2sigma(I)] | R1 = 0.0302, wR2 = 0.0718 | |
| R indices (all data) | R1 = 0.0513, wR2 = 0.0766 | |
| Extinction coefficient | n/a | |
| Largest diff. peak and hole | 2.552 and -1.804 e.$Å^{-3}$ | |
| CCDC Number | 2351162 | |

# References

1. M. Gies, F. Michel, C. Lupó, D. Schlettwein, M. Becker and A. Polity, Journal of Materials Science, 2020, 56, 615–628.

2. E. V. Miu and J. R. McKone, Journal of Materials Chemistry A, 2019, 7, 23756–23761

3. Q. Shi, S. Murcia-López, P. Tang, C. Flox, J. R. Morante, Z. Bian, H. Wang and T. Andreu, ACS Catalysis, 2018, 8, 3331–3342

4. X. Wan, F. Niu, J. Su and L. Guo, Physical Chemistry Chemical Physics, 2016, 18, 31803–31810

5. C. Yin, S. Zhu and D. Zhang, RSC Advances, 2017, 7, 27354–27360

6. U. Prasad, J. Prakash and A. M. Kannan, Sustainable Energy & Fuels, 2020, 4, 1496– 1506
